# Engineering Compact Base Editors by AlphaFold‐Guided Mutation Scan and *Escherichia coli*‐Based Tri‐Selection

**DOI:** 10.1002/advs.202516213

**Published:** 2026-02-03

**Authors:** Ryeo Gang Son, Goeun Kim, Jungjoon K. Lee

**Affiliations:** ^1^ Synthetic Biology for Clinical and Technological Innovation (SynCTI) Synthetic Biology Translational Research Programme and Department of Biochemistry National University of Singapore Singapore Singapore; ^2^ National Centre for Engineering Biology (NCEB) Singapore Singapore; ^3^ Toolgen Seoul South Korea

**Keywords:** base editing, cytidine deaminase, directed evolution, gene therapy, precision genome editing, SsdA_tox_, structure‐guided protein engineering, single‐stranded DNA targeting

## Abstract

APOBEC1‐based cytosine base editors such as BE4max enable base conversion, but many alternative deaminases show low activity and cytotoxicity, especially when miniaturized for delivery. SsdA_tox_, a DNA deaminase toxin from Pseudomonas syringae that is two‐thirds the size of APOBEC1, is attractive for compact base editors but, in native form, shows low C‐to‐T editing efficiency and high cytotoxicity. Guided by an AlphaFold‐ and CASTpFold‐based alanine scan, we identified K31 as a gatekeeping residue whose substitution enlarges the modeled DNA binding pocket. Site‐saturation mutagenesis at K31 produced variants with ten‐fold higher activity but increased indel formation. To further enhance activity while reducing indels and cytotoxicity, we developed Trinity‐Screen, an *Escherichia coli* (*E. coli)*‐based three‐in‐one directed evolution platform that selects for high activity and reduced double‐strand break‐associated indels. Trinity‐Screen revealed four additional DNA‐binding positions; combinatorial mutagenesis at these sites generated four‐ and five‐site SsdA_tox_ variants that retained high activity yet showed lower indel rates and rescued bacterial viability. To standardize comparisons, we defined the Base Editor Performance Index (BEPI), which integrates C‐to‐T conversion and indel frequency. Optimized SsdA_tox_ variants achieved up to 31‐fold improvement relative to wild type, outperforming BE4max at multiple endogenous targets and displaying ten‐fold lower cytotoxicity in *E. coli*.

## Graphical abstract

A miniaturized deaminase SsdA_tox_ was scanned with AlphaFold to identify DNA binding pocket hot spots. Site‐saturation mutagenesis at gatekeeper residue K31 yielded ten‐fold activity enhancement. Trinity Screen, an *E. coli*‐based three‐in‐one platform selecting for high activity and reduced double‐strand breaks, enabled combinatorial evolution at DNA‐binding positions, generating optimized variants with thirty‐one‐fold improvement, lower cytotoxicity, and reduced indels, outperforming BE4max at multiple endogenous targets.

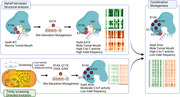

## 1 Introduction

Base editors have transformed the landscape of genome editing by enabling precise single‐nucleotide changes without inducing double‐strand breaks (DSBs), which are often associated with indel formation and cytotoxicity [1, 2, 3]. The first‐generation base editors, built upon the cytidine deaminase APOBEC1 fused to Cas9 nickase, marked a milestone in precision genome editing. While APOBEC‐based systems demonstrated significant utility, their limitations, including size [4, 5], non‐specific DNA editing activity [6], non‐specific RNA editing activity [7], and bystander effect [8] have been reported.

Recently, base editors and prime editors have also been found to induce substantial DSB activities, leading to large deletions in cancer cell lines, human embryonic stem cells, and human primary T cells [9]. These reports underscore the need for alternative base editor enzymes that offer high activity with reduced side effects, including indels caused by DSB activity.

In canonical cytidine base editors, Cas9 nickase introduces a single‐strand nick, while the linked deaminase converts target cytidines to uracil [2, 10]. Subsequent base excision repair and processing of apurinic or apyrimidinic sites can generate double‐strand breaks and indels when nicks occur on opposite strands or when repair intermediates collide during replication [10, 11]. Although deaminases do not directly catalyze DSB formation, their activity determines the number, position, and persistence of uracil lesions, so tuning deaminase properties can indirectly reduce DSB frequency by limiting problematic repair events [10, 11]. Although deaminases do not directly catalyze DSB formation, their activity determines the number, position, and persistence of uracil lesions, so tuning deaminase properties can indirectly reduce DSB frequency by limiting problematic repair events [10, 11]. This mechanistic coupling creates an apparent tradeoff for base editor engineering, because strategies that simply increase deaminase activity typically increase the number of uracil lesions at and around the on‐target site, which in turn raises the probability that opposing strand nicks or replication forks encounter unresolved lesions and generate DSBs. In practical terms, variants with higher on‐target base editing activity usually show higher DSB‐linked indel formation at the same locus, so on‐target activity and DSB formation tend to rise together rather than being independently tunable.

A conceptually similar tradeoff has been observed for SpCas9 nucleases, where efforts to increase specificity often reduce on‐target activity, and across panels of variants and targets, off‐target cleavage generally scales with on‐target cleavage; Cas9 variants that cut very efficiently at their intended site also tend to cleave mismatched off‐target sites more frequently [12]. Building on this activity–specificity framework established in earlier work, we focused on identifying Cas9 variants that escape this constraint and developed Sniper2L, a high‐accuracy SpCas9 variant that behaves as a mutant outlier above the usual tradeoff frontier [13]. Sniper2L preserves wild‐type like on target cleavage while markedly reducing off‐target editing, demonstrating that it is possible to find variants that combine high activity with enhanced specificity rather than conforming to the typical inverse relationship between these properties. Inspired by this precedent, we hypothesized that deaminase variants that sit above an analogous tradeoff frontier could be identified, achieving higher C to T editing efficiencies while at the same time lowering DSB‐associated indel formation within the same base editor.

Deaminases from diverse species, including bacterial toxins and viral proteins, have also been explored as potential substitutes for APOBEC [1, 5, 14, 15, 16, 17, 18]. Despite their functional potential and structural similarity to APOBEC, most native deaminases exhibit low editing efficiency, high indel formation, and substantial cytotoxicity when repurposed for base editing. These challenges have prompted efforts to enhance their performance through rational protein design [16, 19, 20, 21, 22, 23, 24, 25, 26, 27, 28] or directed evolution [1, 4, 29, 30, 31, 32]. Rational design, guided by structural and computational analysis, can pinpoint key residues for mutation to improve enzyme activity or indel formation. Meanwhile, directed evolution, which relies on high‐throughput screening of mutant libraries, can identify beneficial mutations without prior mechanistic knowledge. A combined approach that leverages rational design and directed evolution would offer a powerful solution for systematically improving deaminase enzymes.

In this study, we applied such an integrated strategy to engineer SsdA_tox_ (Single‐strand DNA Toxin A), a small deaminase derived from *Pseudomonas syringae* [33, 34]. SsdA_tox_ is an attractive candidate for base editing due to its compact size, which is advantageous for therapeutic delivery systems, and it has also been demonstrated to be effective in mitochondrial editing [56, 57, 58, 59, 60]. However, the native form of SsdA_tox_ suffers from critical limitations: low editing efficiency, significant cytotoxicity, and a high propensity to induce DSBs and subsequent indels. These drawbacks render it unsuitable for practical base editing applications without extensive optimization [34].

To solve these challenges, we first applied rational design using AlphaFold3 [35] for protein structure prediction, followed by structural and geometric analysis with Molecular Operating Environment (MOE) [36] and CASTpFold [37]. We hypothesized that enhancing SsdA_tox_ activity could be achieved by expanding the DNA‐binding pocket, identified through MOE. To predict mutants with an enlarged DNA binding pocket, we generated a series of single alanine mutants and modeled their structures using AlphaFold3. Although AlphaFold‐family models have transformed structure prediction, multiple reviews and evaluations caution that they do not reliably predict single‐site mutational effects, which often depend on dynamics, ensembles, and context not captured by static models [38, 39, 40, 41, 42, 43]. AlphaFold3‐guided alanine scanning nevertheless helped nominate K31. DNA‐binding pocket areas of these mutants were then evaluated using CASTpFold [37]. The identified key residue (K31) was further optimized through site‐saturation mutagenesis screening.

To further improve the mutant developed by this computational approach, we developed ‘Trinity‐Screen’, a novel *Escherichia coli (E. coli)*‐based directed evolution platform designed for high‐throughput selection of deaminase variants with improved activity, reduced indel formation, and lower toxicity. Through iterative rounds of combination of mutations identified by computational modeling and directed evolution, we systematically optimized SsdA_tox_ to address its native limitations. However, conventional base editor evaluation methods rely on edit/indel ratios, which can lead to misleading assessments. When indel formation rates are low (<1%), even modest editing efficiencies become artificially inflated in the ratio calculation, resulting in disproportionately high weighting of the indel parameter and compromising the accuracy of performance comparisons [44]. To address this limitation, we developed a comprehensive Base Editor Performance Index (BEPI) evaluation system to accurately assess base editor performance by integrating both editing efficiency and indel formation in a more balanced analytical framework as a descriptive single‐amplicon metric complementary to genome‐wide off‐target and bystander‐editing assays. The engineered SsdA_tox_ variants exhibited up to ten‐fold increase in editing efficiency, a two‐fold reduction in indel formation, and ten‐fold reductions in cytotoxicity in *E. coli* compared to the wild‐type enzyme. Importantly, these optimized variants demonstrated comparable or superior performance to BE4max [45], one of the most advanced APOBEC‐based editors, across a variety of sequence contexts.

In summary, we integrate AlphaFold‐guided mutation scan with our innovative three‐in‐one directed evolution strategy to establish a scalable framework for optimizing deaminase enzymes. This approach paves the way for next‐generation base editors with broad applications in both research and therapeutic genome editing.

## 2 Methods

### 2.1 Structure Analysis of SsdA_tox_

Structural analysis was performed using MOE (Molecular Operating Environment, version 2024.0601) [36] with the crystal structure of SsdA_tox_ (PDB ID: 7JTU) and structural models generated by AlphaFold3 [35]. The modeling input included mutant SsdA_tox_ sequences, a Zn^2^^+^ cofactor, and single‐stranded TTCTT DNA. We selected TTCTT as the model DNA sequence rather than alternatives like AACAA to ensure accurate AlphaFold‐based prediction of the spatial arrangement between the deaminase and target cytosine. Since the deaminase in our study specifically targets cytosine residues, the choice of flanking sequence context is critical for reliable structural modeling. During our preliminary analyses, we observed that sequences containing purines (A, G) flanking the target cytosine occasionally resulted in ambiguous or inconsistent predicted binding conformations. This can be attributed to the larger molecular size of purine bases, which can sterically interfere with the spatial positioning of the deaminase active site relative to the target C residue in the predicted structures. The bulkier purine bases may obstruct the optimal alignment between the deaminase and cytosine substrate, leading to less reliable structural predictions.

To minimize this steric interference and obtain more consistent AlphaFold‐predicted structural models, we designed our sequence using only pyrimidine bases (T and C). The TTCTT sequence provides a uniform structural environment that facilitates more accurate and reproducible predictions of the deaminase‐cytosine spatial arrangement across our computational analyses.

Comprehensive inter‐residue distance measurements were conducted using MOE to characterize the spatial organization of key residues within the protein‐DNA complex.

### 2.2 Alanine Scan of SsdA_tox_

Each amino acid position in SsdA_tox_ was individually substituted with alanine, and the resulting structures were predicted using AlphaFold3. For each position, five predicted structures were generated and analyzed separately to calculate the surface area of the DNA binding pocket using CASTpFold [37]. The surface area values were then averaged, and the position with the highest score was identified as the key site.

### 2.3 Construction of pBLC‐SsdACBE Vector

To minimize the cytotoxic effects of SsdA_tox_ in *E. coli*, a low‐copy‐number plasmid (pBLC) used in our previous study [46] featuring the p15A origin was employed for cloning. (High‐copy‐number plasmids were unsuitable due to poor cloning efficiency.) pBLC‐SsdACBE (Figure S1a) was constructed by cloning SsdA_tox_ sequence (Kind gift from Prof. Yongsub Kim [34]) in a base editor format (bpNLS‐SsdA‐nCas9‐UGIx2‐bpNLS) as a substitution for Cas9 in the pBLC‐Cas9 vector [46] using the Gibson Assembly kit (NEBuilder HiFi DNA Assembly Cloning Kit) (New England Biolabs, Ipswich, MA, USA).

### 2.4 Construction of pRep‐sgRNA Vector

The pRep‐sgRNA contains three critical elements: a kanamycin resistance gene with a defective start codon (ACG); an sgRNA targeting the defective start codon, which can correct it (ACG) to the correct codon (ATG) through base editing; and additional sgRNAs targeting repetitive regions of the *E. coli* genome, designed to induce DSBs (Figure S1b). The list of repetitive sgRNAs within *E. coli* genome was obtained by a protocol from Tan et al. [47]. Repeat sgRNAs [*n* = 2 (GTAGGTTCGACTCCTATTAT), 7 (GTCGTCTTCAACGTTCCTTC), or 12 (GCATGGAGCAGATTCTGCCA)] were used to construct pRep2‐sgRNA, pRep7‐sgRNA, and pRep12‐sgRNA, respectively (Figure S1c). The pUC19 vector (Enzynomics, Korea) was used as a backbone to clone the defective kanamycin gene fragment, one sgRNA targeting the start codon of the defective kanamycin gene under the Tet promoter, and another sgRNA targeting the genomic DNA repeat sequence under the T7 promoter. The defective kanamycin was first inserted into pUC19 via Gibson Assembly Kit. A single DNA construct was synthesized, incorporating an sgRNA targeting the *E. coli* genome and an sgRNA targeting the start codon of the kanamycin resistance gene, along with their respective promoter and terminator sequences (Figure S1b). The assembly was performed using BamHI and EcoRI (Enzynomics, Korea) for digestion of pUC19 vector with the defective Kanamycin, followed by ligation with T4 Ligase (Enzynomics, Korea) of the single DNA construct (Figure S1c).

### 2.5 Construction of SsdA_tox_ Library

The SsdA_tox_ library was cloned into the pBLC‐SsdACBE plasmid. The library was constructed using three different strategies. First, error‐prone PCR kits from two different vendors GeneMorph II Random Mutagenesis Kit (Agilent, CA, USA), Diversify PCR Random Mutagenesis Kit (Clontech Laboratories, CA, USA) were used to amplify the SsdA_tox_ fragment, which replaced the WT SsdA_tox_ fragment using the Gibson Assembly Kit (NEBuilder HiFi DNA Assembly Cloning Kit). Error prone PCR was performed using primers flanking the SsdA_tox_ coding region (F‐ 5′ TCACCAAAGAAGAAGCGGAAAGTCAAGCTT 3′, R‐ 5′ GCCGGAGCTGCCACCGGAGGAGCCTCCGCT 3′), such that only the deaminase domain was mutagenized while the linker and Cas9 regions remained unchanged. We utilized the XL1‐Red mutator strain (Agilent, CA, USA) to generate a library of SsdA_tox_ mutants through error‐prone replication. For the mutant strain, the library was constructed by transforming the pBLC‐SsdACBE plasmid into the XL1‐Red mutator strain and incubating it according to the vendor's protocol. The third approach involved amplifying the mutant SsdA_tox_ templates obtained from XL1‐Red using an Error‐prone PCR kit.

For quality control of the naive SsdA_tox_ library, the deaminase coding region was amplified from the pooled pBLC‐SsdACBE plasmid library using primers flanking the SsdA_tox_ open reading frame and subjected to Illumina paired‐end sequencing. Read pairs were merged and aligned to the reference SsdA_tox_ coding sequence, and mutation profiles were quantified with CRISPResso2 using default parameters and a quantification window spanning the entire coding region. The mutation position distribution plot reported the percentage and absolute number of reads with substitutions, insertions, or deletions at each nucleotide position, providing an overview of library diversity and positional uniformity before Trinity‐Screen selection.

### 2.6 Codon Optimized Site‐Saturation Mutation for K31 Position

All site‐saturation mutants of SsdA_tox_ were generated for the K31 position using a novel codon design strategy that allows uniform expression of all 20 amino acids using tailored codons (VAS/DGG/TNC/VYC/ATG) to encode 20 amino acids (HQNKDE/WRG/FSYC/LPITVA/M) with precise 1:1 ratio. Unlike conventional NNK, NNS (N = A/T/G/C; K = T/G and S = G/C) or c‐22 trick methods, our approach eliminates rare codon usage, ensuring optimal expression in mammalian cells and addressing a critical limitation in existing techniques [48]. We developed mutant constructs by mixing primers containing five codon types (VAS/DGG/TNC/VYC/ATG) in a 6:3:4:6:1 ratio. To construct the SsdA_tox_ mutant, we selected the AflII site located between the N‐terminal bpNLS and SsdA_tox_, and the BglII site located at 250 bp downstream of the N‐terminus of Cas9. The first DNA fragment was amplified by PCR from 28 bp upstream of the AflII site to 20 bp downstream of the target mutation point. The second DNA fragment was amplified from 20 bp downstream of the target mutation point to 30 bp downstream of the BglII site, incorporating overlapping regions for Gibson assembly. The two fragments were assembled into the AflII/BglII‐digested pBLC‐SsdACBE vector using Gibson assembly. The assembled plasmids were transformed into DH5α cells (Enzynomics, Korea), and plasmid DNA was purified using a mini prep kit (Favorgen Biotech, Taiwan) and a endotoxin‐free midi prep kit (Geneall, Korea).

### 2.7 Development of BEPI Evaluation System

To enable comprehensive base editor assessment integrating both editing efficiency and indel formation, we systematically designed fifteen mathematical formulations with varying penalty structures (Table S2). We developed formulas across four mathematical frameworks: (1) arithmetic operations, (2) rational functions, (3) logarithmic scaling, and (4) exponential decay models. Each formula was tested using C‐to‐T rates (2.5%–100%) and indel frequencies (0%–10%) across 56 performance scenarios. Formulas were assessed for computational stability, sensitivity patterns, and practical utility to identify the optimal evaluation system.

### 2.8 Trinity‐Screen

Trinity‐Screen was developed to select SsdA_tox_ variants that combine high base editing activity with low DSB and low cytotoxicity within a single *E. coli* workflow. Electrocompetent *E. coli* BW25141 cells carrying a pRep‐sgRNA were transformed with the pBLC‐SsdACBE library (10 ng) using a Bio‐Rad GenePulser II set at 2.5 kV and plated on LB agar supplemented with kanamycin. On these plates, Trinity‐Screen applies three simultaneous selection pressures. In the activity selection axis, cells expressing active SsdA_tox_ variants convert the defective ACG start codon of the kanamycin resistance gene to ATG and survive. In the DSB reduction axis, sgRNAs target highly repetitive genomic loci so that variants with high DSB rates generate lethal double‐strand breaks and are eliminated. In the cytotoxicity reduction axis, variants that are intrinsically highly toxic cannot tolerate continuous expression and fail to form colonies. Thus, surviving colonies have, by definition, passed all three selection axes at once. Plasmids were extracted from surviving colonies using a mini prep kit. The extracted plasmids contained both the pRep‐sgRNA and the pBLC‐SsdACBE variants. To isolate pBLC‐SsdACBE plasmids, a restriction enzyme was used to specifically cleave and inactivate the pRep‐sgRNA while leaving pBLC‐SsdACBE intact. After restriction digestion, pBLC‐SsdACBE plasmids were purified and retransformed into fresh BW25141 cells carrying the pRep‐sgRNA for the next round of selection. This four‐round process was performed with decreasing amounts of plasmid introduced in each successive transformation to increase stringency. After the final round, plasmids from surviving colonies were pooled, and targeted Illumina sequencing was used to quantify the copy number of each mutant, providing a quantitative measure of enrichment across the library. The independent DH5α transformation assay described in the toxicity section, in which individual SsdA variants are expressed without the pRep‐sgRNA, was carried out only after Trinity‐Screen to validate intrinsic toxicity and was not part of the selection itself.

Sequencing data from the first to the fourth rounds were analyzed using the following method. Read2 was reverse‐complemented using Biopython, and pairwise alignment was performed to overlap Read1 and Read2 for sequence reconstruction. The reconstructed sequences were cumulatively counted to determine the read count results. Each sequence was labeled with the corresponding round and read count, and mutants were sorted using XLibraryDisplay [49]. Sequences that exhibited more than a 1000‐fold increase in abundance in the fourth round compared to the first round were selected for gene synthesis.

### 2.9 Toxicity Test of Mutant SsdA_tox_ in *E. coli*

To assess the intrinsic toxicity of SsdA_tox_ independently of any selection pressure, 50 µL of chemically competent DH5α cells (NEB) were transformed at 42°C with pBLC‐SsdACBE variants (10 ng) in the absence of the pRep‐sgRNA. The cells were incubated in 200 µL SOC medium (NEB) for 1 hour, and 30 µL was plated on LB plates containing chloramphenicol, and incubated at 37°C overnight. The number of colonies for the mutants was compared with that of the WT after counting using NIST's Integrated Colony Enumerator (NICE) software [50].

### 2.10 Base Editing in Mammalian Cells

To facilitate SsdA_tox_ evaluation, a model HEK293T cell line was established. This line was stably integrated with 24 sgRNA and target pairs to allow a comprehensive assessment of base editor activity (Table S1). These sgRNAs targeted regions with varying base editing configurations, including cases with no cytosines (mono‐C), single isolated cytosines (mono‐C), two consecutive cytosines (di‐C), and three consecutive cytosines (tri‐C).

To construct the library, the oligos (listed in Table S1), containing sgRNA and target pairs, were individually synthesized (Twist Bioscience, CA, USA) and cloned into a PiggyBac vector under the U6 promoter [51]. HEK293T stable cell lines were generated by transfecting the PiggyBac library and PiggyBac transposase plasmids (System Biosciences, CA, USA) using a standard transfection protocol using Lipofectamine 2000 (Thermo Fisher Scientific, Waltham, MA, USA), followed by puromycin selection.

Each stable cell line was seeded at 1 × 10^5^ cells per well in a 24‐well plate and transfected with 1 µg of pBLC‐SsdACBE variants using Lipofectamine 2000 (Thermo Fisher Scientific, Waltham, MA, USA). Media was replaced 16 hour post‐transfection, and cells were harvested 48–60 hour post‐transfection for optimal confluency (∼90%) to ensure accurate assessment of activity and indel rates. Genomic DNA (gDNA) was extracted from the harvested cells using DirectPCR Lysis Reagent (Cell) (Viagen Biotech, Cedar Park, TX, USA) and Protease K (Sigma‐Aldrich, St. Louis, MO, USA) according to the manufacturer's protocol. NGS sequencing with Illumina Nextseq 150 × 2 cycle kit was employed to analyze editing outcomes in HEK293T cells. Editing efficiency and indel formation rates were calculated using the RGEN Base Editor analyzer (http://www.rgenome.net/be‐analyzer/#!) [52]. The BE‐analyzer was utilized under the following conditions: Illumina paired‐end FASTQ files were used as input. The reference sequence was constructed by including the barcode sequence corresponding to each EMsg following the U6 terminator sequence (CTTTTTT), followed by the GA sequence, the EMsg sequence, and the PAM (Table S1). This was then followed by a fixed sequence, AGCTTGGCGTAACGGCTTAACTAGATTAAT. The editing window range was set between 11 and 19, while all other parameters were kept at default settings.

In BE Analyzer, the C‐to‐T editing efficiency is calculated exclusively for cytosines within the user‐defined analysis window; this does not necessarily correspond to the intrinsic editing window of the base editor protein itself. Due to the low baseline activity of wild‐type SsdA, we were unable to determine its precise editing window a priori, nor could we predict how mutations would alter the window boundaries during our directed evolution campaign.

To ensure comprehensive data collection without introducing bias, we specified a broad analysis window (positions 11–19) that would capture editing events across a wide protospacer range. Post‐analysis validation confirmed that the actual editing windows of both SsdA variants and BE4max fell entirely within this pre‐defined range. This approach allowed us to report reliable C‐to‐T editing efficiencies without excluding potentially relevant editing positions, while the actual editing window characteristics for each variant were determined empirically from the resulting data distributions.

Indel frequency was defined as the percentage of sequencing reads containing insertions or deletions overlapping the protospacer and PAM region, divided by the total number of aligned reads at that locus.

### 2.11 Validation of SsdAtox Variants in Endogenous Target

10 different endogenous target loci were selected, and their sgRNAs were designed from Kweon et al. [34]. The sgRNAs were cloned into pRG2 plasmid.

The pRG2‐sgRNA (100 ng) and pBLC‐SsdACBE vectors (100 ng) were co‐transfected into HEK293T seeded 1.6 × 10^4^ cells per well in 96 well using Lipofectamine 2000.

The remaining protocols for genomic DNA isolation using DirectPCR Lysis Reagent (Cell) and NGS analysis are the same as described in the previous section, using primers targeting each endogenous locus from Kweon et al. [34].

### 2.12 NGS Analysis

NGS samples were prepared using a three‐step PCR process comprising target amplification in the first PCR, adaptor attachment in the second PCR, and index incorporation in the third PCR.

The Illumina NGS 300 × 2 cycle kit was used with the Miseq sequencing system (Illumina, CA, USA) to identify SsdA_tox_ mutants, while the 150 × 2 cycle kit with the Nextseq 1000 sequencing system (Illumina, CA, USA) was employed to evaluate base editing efficiency and indel formation.

## 3 Results

### 3.1 Construction and Optimization of SsdA_tox_ Vectors as Cytosine Base Editor

We selected the recently identified SsdA_tox_ as a model deaminase for optimizing the deaminase component of a base editor [34]. While SsdA_tox_ shows many beneficial aspects compared to the Apobec1, such as a smaller size (151 amino acids) than rApobec1 deaminase domain (229 amino acids), its wild‐type version showed significantly lower base‐editing activity, high indel frequency, as well as high cytotoxicity. We reasoned that this system is potentially suitable for testing our optimization methodology. To reduce the cytotoxic effects of SsdA_tox_ in *E. coli* [34], we utilized a low‐copy‐number plasmid backbone (pBLC) with the p15A origin, previously described in our earlier study [46]. We cloned our SsdA_tox_ sequence to substitute Apobec1 sequence of the BE4max plasmid to construct pBLC‐SsdACBE (Cytosine Base Editor) plasmid (Figure S1a). To systematically test mutants across a larger number of targets, we constructed a HEK293T cell library containing 24 sgRNA and target pairs embedded in its genomic DNA using PiggyBac transposase (Table S1). This constructed library, named Exogenous Model sgRNAs (EMsgs), enabled comprehensive evaluation of editing patterns across diverse target sequences.

### 3.2 Development of BEPI for Base Editor Assessment

A key challenge in base editor optimization is the inherent trade‐off between editing efficiency and safety, as increased C‐to‐T conversion often coincides with elevated indel formation. To address this, we developed a new metric, termed the BEPI, designed to quantify and compare the performance of base editors by jointly considering editing efficiency and associated indel rates. An effective BEPI should satisfy three core criteria: (1) balanced sensitivity to both low and high indel frequencies; (2) robust discrimination between base editors across a range of C‐to‐T conversion efficiencies; and (3) the capacity to reward high editing efficiency even when indel frequencies are proportionally elevated, reflecting realistic trade‐offs encountered during optimization.

To fulfill these criteria, we constructed fifteen mathematical formulations of the BEPI (Table S2) and systematically evaluated their performance across a matrix of simulated scenarios: seven C‐to‐T conversion levels (ranging from 2.5% to 100%) and eight indel frequency levels (ranging from 0% to 10%), yielding 56 data points per formula (Figure S2). Although sigmoid‐based functions may appear intuitive for modeling progressively increasing indel penalties, we did not adopt such formulations at the outset because their mathematical properties introduce substantial drawbacks for constructing a consistent evaluation metric. Inverted logistic shapes, in particular, provide limited discriminative power in the low‐indel region due to an inherent plateau, and exhibit abrupt collapses at mid‐to‐high indel frequencies (convergence zones), where small changes in indel produce disproportionately large differences in score. These behaviors strongly depend on arbitrary scaling constants, making the resulting metric highly sensitive to parameter tuning and difficult to apply consistently across different experimental settings. For these reasons, sigmoid‐based penalty functions were excluded from our initial design space. Evaluation of the formulations revealed distinct performance profiles. Formulas 2, 3, 4, 5, 6, and 8 exhibited excessive penalization of indels, disproportionately lowering scores even at minimal indel levels. In contrast, Formulas 1, 7, and 9 showed limited sensitivity to indels, primarily reflecting C‐to‐T efficiency and failing to capture meaningful variation in indel rates. More balanced behavior was observed in formulations that integrated both components effectively (Figure S3). For instance, base editors achieving 75% C‐to‐T conversion with 7.5% indels were appropriately scored higher than those achieving 50% conversion with 5% indels, an outcome consistent with performance prioritization in high‐efficiency contexts.

This weighting scheme prioritizes performance in high‐efficiency contexts by assigning greater penalties to indels at higher C‐to‐T rates, conceptually similar to approaches that evaluate base editor behavior across efficiency and safety dimensions in mammalian systems [10, 53]. In pharmacology, related principles underlie optimization of the therapeutic index, where high potency candidates are preferred because they permit dose minimization and thereby reduce toxicities and treatment burden [54, 55]. By analogy, favoring base editing conditions that achieve high on‐target cytosine conversion with low indel formation in high‐efficiency regimes is consistent with the goal of minimizing downstream risks and resource demands associated with less efficient editing configurations. Among the fifteen models, Formula 11 (C‐to‐T / exp(Indel/10)) initially showed promise by accurately comparing editors with similar activity‐to‐indel ratios, particularly at high efficiency. However, it underperformed in low‐efficiency settings due to reduced responsiveness to indels. We therefore identified Formula 14, defined as (C‐to‐T − Indel) / exp(Indel/10), as the optimal formulation. Formula 11 (C‐to‐T / exp(indel / 10)) and Formula 14 ((C‐to‐T − indel) / exp(indel / 10)) share the same exponential indel penalty term, which accounts for their nearly overlapping response surfaces across the typical range of C‐to‐T and indel values. The limitation we highlight arises in Formula 11 because, at low indel frequencies, the exponential factor exp(indel / 10) increases only gradually, so the resulting penalty is shallow, and discrimination between conditions with 0 percent and low indel levels remains weak. Formula 14 mitigates this issue by introducing a direct linear penalty in the numerator (C‐to‐T − indel), which substantially improves separation between 0 percent and low indel frequencies while retaining the same exponential scaling at higher indel burdens. Formulas 11 and 14 therefore, share a common structural basis, yet Formula 14 provides more informative discrimination across the full indel spectrum, particularly in low indel regimes that are of greatest interest for therapeutic optimization.

### 3.3 Identification of K31X Mutation via AlphaFold Alanine Scan and Site Saturation Mutation Screen

**FIGURE 1:**
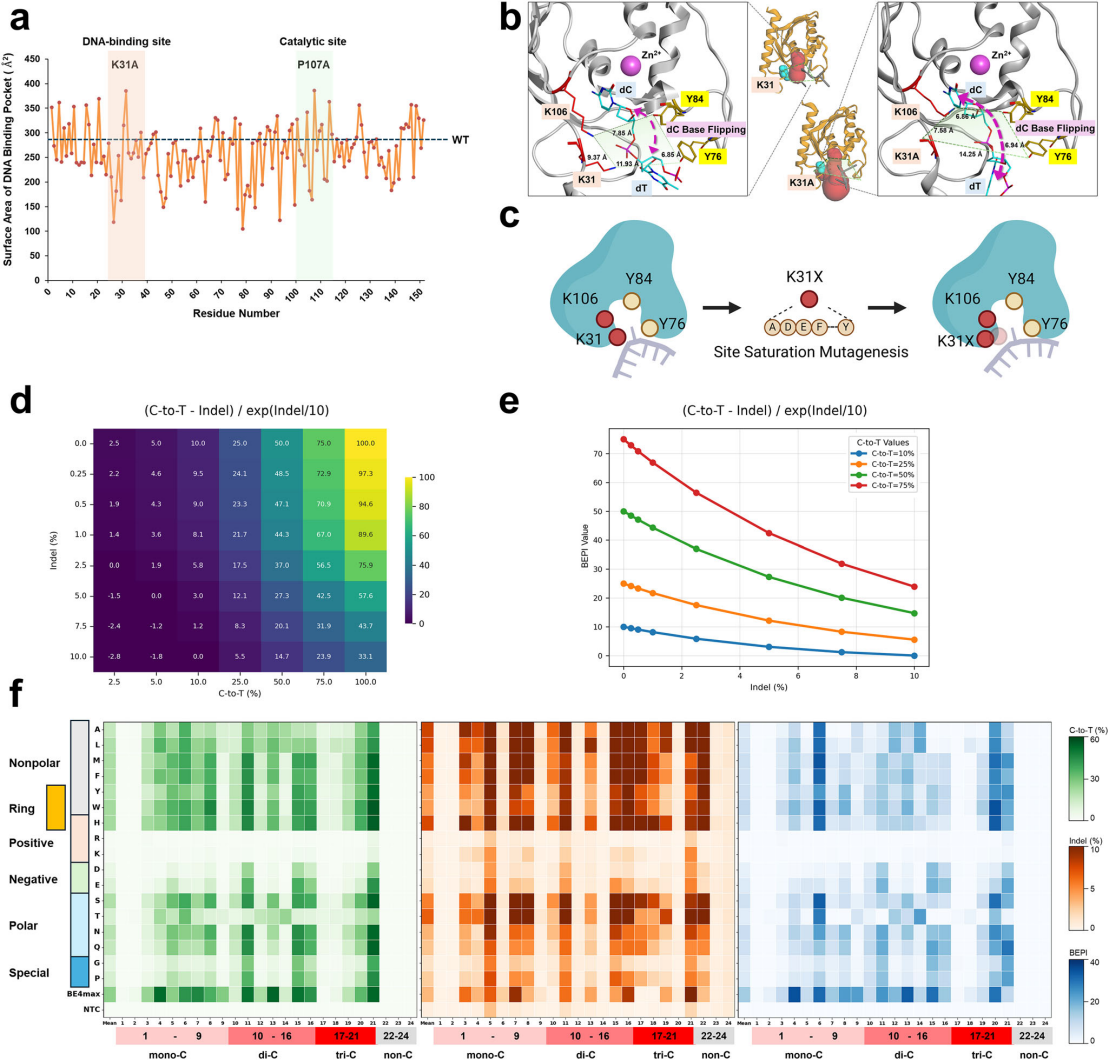
AlphaFold generated structural analysis and its mutant evaluation. (a) Alanine scanning results showing amino acid positions within SsdA_tox_ and surface area of the DNA binding pocket. Red dots represent mean surface area values from 5 structural predictions. (b) Structural analysis between WT SsdA_tox_ and single strand DNA. The DNA binding pocket identified by CASTpFold is shown in red. K31 and K31A residues are highlighted in blue. The enlarged view shows the spatial arrangement of key gatekeeping residues (K31, Y76, Y84, K106) forming the active site entrance (green), with the target cytidine substrate (blue) positioned for base flipping. Purple arrows indicate the base flipping pathway of cytidine into the active site. (c) A Schematic illustration of the hypothesis that the pocket size can be changed by replacing K31 of SsdA_tox_ with another amino acid. (d) Heatmap showing BEPI values across C‐to‐T editing rates (2.5%–100%) and indel frequencies (0%–10%) for the selected formula. Yellow indicates higher BEPI values (better performance), while blue indicates lower values. (e) Sensitivity analysis showing how the selected BEPI formula responds to increasing indel frequency (0%–10%) at different C‐to‐T conversion levels (10%, 25%, 50%, 75%). The formula effectively balances editing efficiency against unwanted indel formation across diverse experimental conditions. (f) Evaluation C‐to‐T substitution rate, indel rate, and BEPI of K31X via 24 EMsgs in HEK293T (*n *= 2). The first column in the heatmap represents the mean values across the 24 EMsgs.

We first analyzed the structure of the SsdA_tox_ protein [Protein Data Bank (PDB) ID: 7JTU] using MOE and identified its active site as well as the DNA binding site. We hypothesized that the narrow DNA‐binding site could potentially be the reason for its low activity, which may be enhanced through rational design to increase the pocket size. To select the amino acid residue sensitive to the pocket size, we performed alanine scanning by predicting the structures of SsdA_tox_ mutants with single alanine substitutions. The surface area and volume of the DNA binding pocket in the predicted structures by AlphaFold3 were calculated using CASTpFold [37]. Upon ranking the results, the K31 position was identified as a key residue influencing pocket size, an increase of which may lead to increased activity (Figure 1a).

Structural predictions using AlphaFold3 revealed that SsdA_tox_ contains a lysine‐rich pocket around the K31 residue, which interacts with the DNA phosphate backbone. In the predicted structure of SsdA_tox_ bound to a DNA substrate (TTCTT) and Zn^2^^+^ ion, spatial distances were measured as follows: 9.37 Å between K31 and K106, 11.93 Å between K31 and Y76, 7.85 Å between K106 and Y84, and 6.85 Å between Y76 and Y84. These four residues form a cluster at the entrance of the DNA binding pocket (Figure 1b) and appear to serve as gatekeepers, facilitating the base‐flipping and selection of target cytosines. Although K31 does not directly participate in the catalytic deaminase reaction, it may act as a spacer that repositions adjacent bases, enabling optimal placement of cytosine within the active site. The positive charge of K31 likely contributes to stabilizing the interactions with the ssDNA phosphate backbone. Based on these structural features, the interaction between K31 and the DNA phosphate backbone may constrict the narrow entrance of the binding pocket, potentially impeding access of cytosine substrates with lower intrinsic deaminase activity. In contrast, the K31A mutation eliminates the positive charge, disrupting its interaction with the DNA phosphate backbone. Structural modeling shows that this substitution increases the distance between K31A and Y76 to 14.25 Å (compared to 11.93 Å in wild‐type), effectively widening the DNA binding pocket entrance. This expansion may facilitate more efficient base‐flipping and improved access of target cytosines to the active site (Figure 1b).

We hypothesized that removing the positive charge through site‐directed mutagenesis of K31 would displace the side chain, creating additional space within the DNA binding pocket (Figure 1c). Consistent with this hypothesis, structural models generated for site‐saturation mutants using AlphaFold3 showed that substitutions at K31, except for lysine (K), arginine (R), and histidine (H), all of which are positively charged, resulted in side chain displacement, thereby enlarging the DNA binding pocket.

Next, we constructed base editor constructs with site‐saturation mutation in K31 position, excluding K31I and K31V variants, which could not be cloned due to toxicity, and K31C variant to prevent unwanted disulfide bond formation and cysteine‐metal ion interactions. Based on simulation models encompassing the theoretical range of base editor activity and indel formation (Figure 1d), the SsdA variant's performance was evaluated using BEPI, which summarizes how combinations of C‐to‐T conversion and indel frequency relate to the composite score under these conditions (Figure 1e). When evaluated using BEPI calculations, the performance ranking was BE4max > W > N > M > Y > F > Q > S > H > L > A > P > E > G > T > D > K > R (Figure 1f). SsdA_tox_ base editors carrying K31X substitutions were tested in HEK293T cells with a panel of 24 exogenous sgRNAs (17 variants across 24 guides). Across this panel, several K31X variants showed higher C to T editing than wild type SsdA_tox_, and the best variants (K31W, N, M, Y) reached up to a 30‐fold increase in C to T frequency on the specific target (Figure 1f). SsdA_tox_ base editors with K31X mutations showed enhanced C‐to‐T editing efficiency up to 30‐fold compared to WT SsdA_tox_ at specific target sites, with W, N, M, Y, F, and Q variants achieving the highest improvements. Several variants achieved C‐to‐T editing levels comparable to BE4max. Additional site saturation mutagenesis revealed that WT Lysine (K) and Arginine (R) produced minimal activity (∼1%), consistent with the hypothetical inhibitory role of positive charges at this position. Substitutions with hydrophobic (A, L, M, F), ring‐structured (H, Y, W), and polar (N, Q, S, T) residues led to >ten‐fold increases in editing efficiency on average. However, these gains were coupled with higher indel rates, reflecting the trade‐off between activity and indel formation.

### 3.4 Directed Evolution With Trinity‐Screen

**FIGURE 2:**
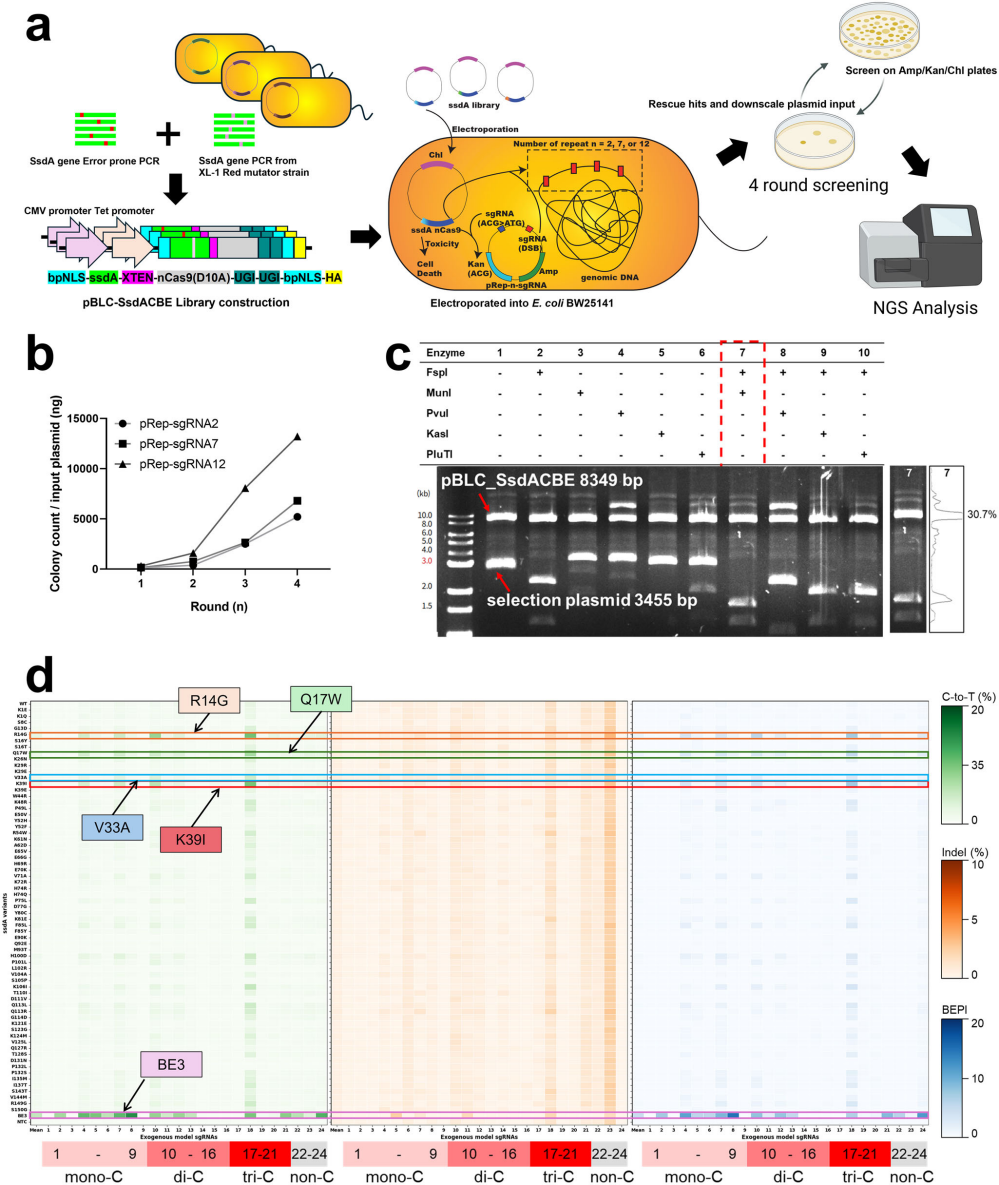
Trinity‐Screen and hit evaluation. (a) Schematic overview of the Trinity Screen workflow. Electrocompetent *E. coli* BW25141 cells that carry pRep‐sgRNA encoding a defective kanamycin resistance gene and two sgRNAs are transformed with a pBLC SsdACBE variant library and plated on kanamycin. On these plates, each cell simultaneously experiences three selection pressures: Rescue of the defective kanamycin start codon by base editing, elimination of variants that generate DSBs at repeated genomic targets, and removal of highly cytotoxic SsdA_tox_ variants that cannot tolerate continuous expression. Surviving colonies from each round provide plasmids for the next round and for deep sequencing‐based hit identification. (b) Summary of Trinity Screen progress, showing for each pRep‐sgRNA the number of colonies that survive all three selection axes at a given plasmid DNA input. Increases in colony number. Across rounds reflect the combined effects of reduced toxicity and improved activity and indel profiles. Quantitative enrichment of individual SsdA_tox_ plasmids was assessed by deep sequencing the input library and the pool recovered. After the fourth round. The three pRep‐sgRNAs use repetitive genomic targets that contain editable cytosines at positions 13 (pRep2‐sgRNA), 12 and 15 (pRep7‐sgRNA), and 12 (pRep12‐sgRNA) relative to the PAM, with the n = 12 for pRep12 plasmid compensating for the weaker activity observed at position 12 in Figure S10. (c) Isolation of pBLC‐SsdA_tox_ plasmid without gel purification via enzyme digestion of the kanamycin gene recovered from pRep‐sgRNA. Band intensities of the recovered plasmids were quantified using ImageJ. (d) Activity screening of SsdA_tox_ variants identified by Trinity‐Screen Against 24 EMsg sites in HEK293T cells (*n *= 1). NTC indicates the Lipofectamine‐only control in which all transfection procedures were performed identically, except for plasmid addition.

Despite the high base‐editing activity of the K31X variants, they also exhibited elevated indel rates and marked toxicity. Prior directed‐evolution platforms for base editors have largely optimized activity alone, without explicitly suppressing indel formation or penalizing toxicity [1, 4, 29, 30, 31, 32]. We previously developed the two‐in‐one Sniper‐Screen for SpCas9 nucleases, which enriches variants that retain on‐target DSB activity while disfavoring off‐target cleavage [13, 46]. Here, we extend that paradigm with a three‐in‐one, *E. coli*–based Trinity‐Screen that replaces the off‐target discrimination axis with direct selection against DSB, while simultaneously selecting for high base‐editing activity and reduced toxicity (Figure 2a). Trinity Screen applies three concurrent selection pressures in a single *E. coli* workflow: activity selection via rescue of a mutated kanamycin resistance start codon, DSB selection at repetitive genomic sites, and cytotoxicity selection based on the ability of cells to tolerate continuous expression of SsdA_tox_ variants during the selection cycle. As in any iterative selection scheme, repeated rounds will favor variants with lower intrinsic toxicity, but here we treat toxicity as an explicit third selection axis that is independent of activity gain and indel suppression rather than as an automatic consequence of improved activity.

Mechanistically, Trinity Screen exploits the fact that uracil bases introduced by SsdA_tox_ at repetitive genomic loci in Escherichia coli are excised by uracil DNA glycosylase (UNG) to generate abasic sites, which are then incised predominantly by the major AP endonucleases Exonuclease III and Endonuclease IV [56, 57, 58, 59, 60]. Processing of clustered uracil and abasic lesions through this pathway is known to yield lethal chromosomal double‐strand breaks in *E. coli*, particularly when repair nicks on one strand are closely opposed to a nick on the opposite strand [56, 57]. It was also reported that inhibition of UNG reduces DSB activity, underscoring the central role of UNG initiated base excision repair in generating these lesions [61]. Therefore, variants that drive extensive deamination at the repetitive targets experience a higher burden of DSBs and are selectively eliminated in the Trinity Screen. At the same time, because UNG activity is needed for conversion of uracil lesions into abasic sites and DSBs, it is conceivable that higher binding affinity of SsdA_tox_ with uracil could partially attenuate UNG function, analogous to uracil glycosylase inhibitors. In this scenario, SsdA_tox_ variants could occupy an intermediate regime in which increased deamination raises DSB formation, whereas increases in binding affinity to uracil product might begin to dampen UNG‐dependent processing and reduce DSB yield. Although we do not resolve these possibilities here, our selection design ensures that the variants recovered by Trinity Screen are those that support sufficient on‐target editing at the selection cassette while avoiding lethal levels of DSB formation at repetitive genomic sites.

To implement the activity and DSB‐linked selection axes, we mutated the start codon of the kanamycin resistance gene from ATG to ACG and co‐expressed a single guide RNA targeting the ACG codon, so that only cells expressing active SsdA_tox_ variants that convert ACG to ATG survive on kanamycin plates. In parallel, we cloned a second sgRNA targeting highly repetitive genomic sites in *E. coli*, following the strategy of Tan et al. [47]. The three pRep(2, 7, 12)‐sgRNA contained repeat targets present two, seven, or twelve times in the genome (pRep2‐sgRNA, pRep7‐sgRNA, and pRep12‐sgRNA, respectively, Figure S1c). In pRep2‐sgRNA, the editable cytosine lies at position 13 relative to the PAM, whereas pRep7‐sgRNA contains cytosines at positions 12 and 15, and pRep12‐sgRNA contains a cytosine at position 12. The high repeat copy number in pRep12‐sgRNA therefore, compensates for this weaker single site activity and ensures that variants that still support low‐level editing at position 12 generate enough DSBs at genomic repeats to be efficiently removed. In principle, any iterative selection will tend to reduce overall toxicity; here we explicitly design and report toxicity as a third axis, separate from activity gain and indel suppression, to make this effect transparent.

Moreover, the inherent toxicity of SsdA_tox_, evidenced by the low number of surviving colonies containing the SsdA_tox_ vector, provided further selective pressure (Figure 2b). Overall, surviving colonies harbored mutant SsdA_tox_ variants with enhanced activity, reduced indel formation, and lower toxicity.

The SsdA_tox_ library was constructed by cloning the pBLC‐SsdACBE plasmid using three strategies: (1) Error‐prone PCR with kits from GeneMorph II and Diversify PCR to amplify and replace the WT SsdA_tox_ fragment via Gibson Assembly; (2) Error‐prone replication in the XL1‐Red mutator strain by transforming the plasmid and incubating as per protocol; and (3) amplification of mutant SsdA_tox_ templates from XL1‐Red using an Error‐prone PCR kit.

Before applying Trinity‐Screen, we combined three types of libraries and assessed the composition of the combined pBLC‐SsdACBE plasmids. Illumina amplicon sequencing followed by CRISPResso2 analysis showed that the library contained substitutions distributed across the full length of the SsdA_tox_ coding region, with typical per‐base substitution frequencies of approximately 1% to 2% and occasional hotspots reaching about 5% to 6%, and with insertions and deletions remaining rare at most positions (Figure S4a). This pattern indicates that the mutagenesis procedures produced a broadly sampled library without extended regions devoid of mutations and that the initial pool entering Trinity‐Screen was not dominated by a small subset of sequence variants.

Electrocompetent *E. coli* BW25141 cells containing pRep‐sgRNA were transformed with the pBLC‐SsdACBE library via electroporation and plated on LB agar supplemented with kanamycin (25 µg/mL), ampicillin (50 µg/mL), and chloramphenicol (12.5 µg/mL). Surviving colonies endured the following selection pressures: (1) Activity selection, enabling kanamycin resistance through active SsdA_tox_ variants; (2) indel reduction selection, eliminating variants with high indel rates caused by *E. coli* cell death by sgRNA‐induced DSBs; and (3) Cytotoxicity reduction selection, removing toxic variants. The resulting colonies displayed reduced cytotoxicity, lower indel formation, and improved base editing activity. The selection process was repeated over four rounds (Figure 2b), progressively enriching SsdA_tox_ variants with favorable activity, indel, and cytotoxicity profiles.

After each round of selection, the pBLC‐SsdACBE plasmid library needed to be isolated. However, the surviving colonies contained both the mutant pBLC‐SsdACBE plasmid and the pRep‐sgRNA. To improve the efficiency of isolating the pBLC‐SsdACBE plasmid from the plasmid mixture, we recovered the pBLC plasmid without performing gel extraction. To achieve this, we designed a list of restriction enzymes specifically targeting the digestion of the pRep‐sgRNA (Figure S4b). We confirmed that the combination of FspI, which disrupts kanamycin and ampicillin resistance through blunt‐end cutting, and MunI, which cleaves the sgRNA region with cohesive ends, is the most effective for eliminating the pRep‐sgRNA (Figure 2c).

### 3.5 Hit Identification and Site Saturation Mutagenesis

After four rounds of Trinity‐Screen, 67 unique single mutations were identified by NGS analysis. Among them, four mutants (R14G, Q17W, V33A, and K39I) were identified for their enhanced base editing activities when they were assessed with EMsgs (Figure 2d). These mutants exhibited a two‐ to four‐fold increase in C‐to‐T editing efficiency compared to SsdA_tox_ WT, although their indel rates were similar to or slightly higher than those of WT. Mutants showed particularly high activity with sgRNAs 11, 15, 16, and 21, while sgRNAs 4, 5, and 21 induced significantly higher indel rates. This pattern differed markedly from APOBEC1 of BE3, suggesting that SsdA_tox_ recognizes different target sequence contexts.

Notably, all four identified critical positions (R14, Q17, V33, K39) are located within the DNA‐binding domain, consistent with our initial K31X variants that also reside in this region. This clustering pattern suggests that DNA‐binding site modifications represent a primary mechanism for enhancing SsdA_tox_ activity, likely through improved substrate recognition or positioning. Additionally, beneficial mutations were detected near the active site around P107, indicating that catalytic domain optimization also contributes to enhanced performance. However, given the concentrated emergence of effective mutations within the DNA‐binding region and the proven success of our K31X variants, we focused our optimization strategy on DNA‐binding site modifications rather than active site engineering.

Interestingly, despite K31X variants showing superior activity in initial screening, they were not recovered in the final Trinity‐Screen results. NGS analysis revealed that K31N was present in the library pool and persisted through the second round without significant enrichment, but was subsequently eliminated, confirming that high‐indel mutants were selectively removed during the screening process. This selective elimination validates our Trinity‐Screen design, which effectively identifies mutations that enhance activity while maintaining acceptable indel profiles.

### 3.6 Combination and Evaluation of SsdA4mix Variants

**FIGURE 3:**
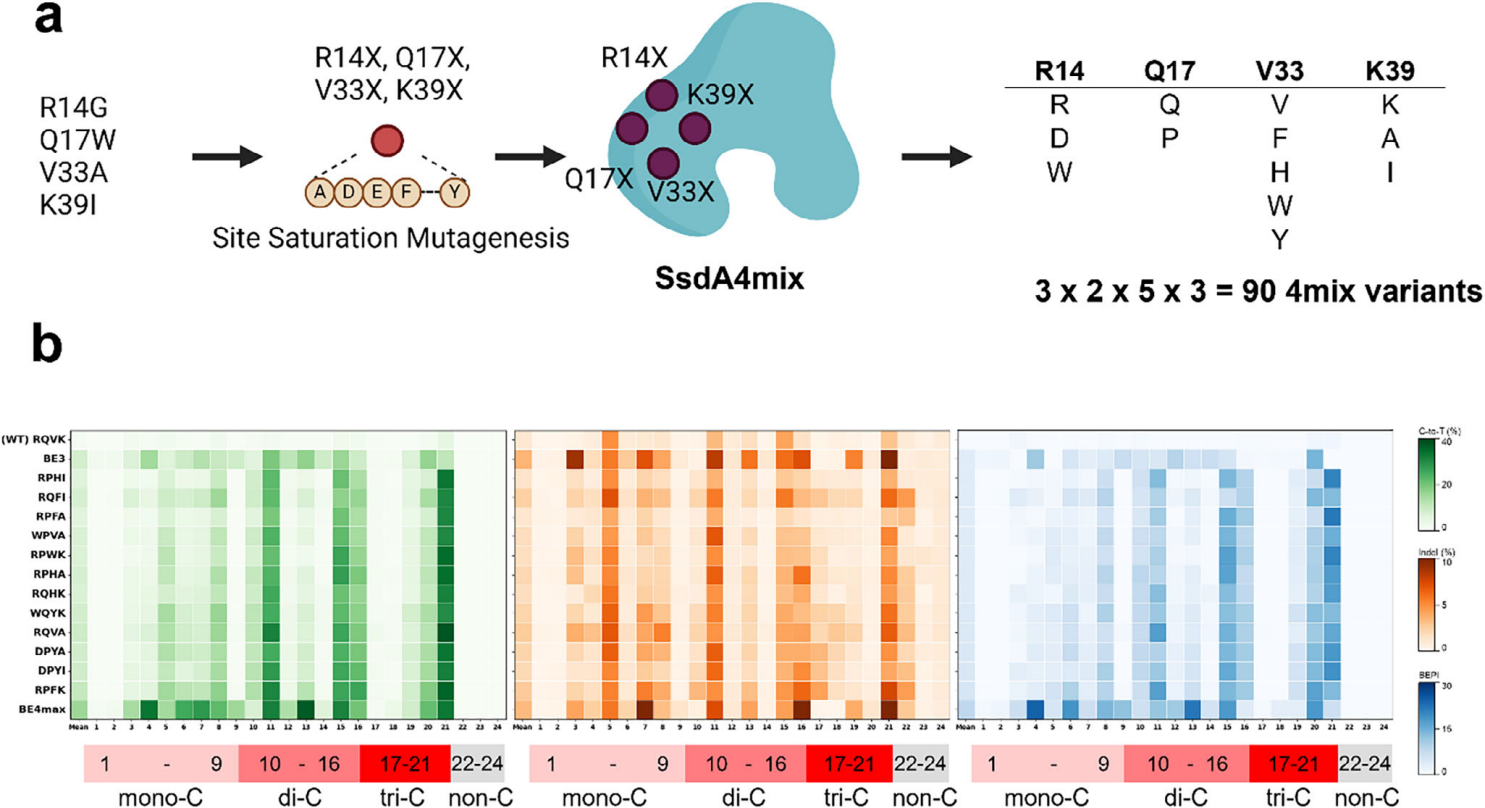
Combinatorial mutagenesis of SsdA4mix and its assessment. (a) Schematic workflow for SsdA4mix development. Trinity‐Screen identified four key mutation sites, followed by site saturation mutagenesis at these positions. Hit variants from each site were subsequently combined to generate SsdA4mix candidates for further analysis. (b) Assessment of SsdA4mix variants using 24 EMsgs in HEK293T cells. WT is shown as reference, followed by selected variants ranked by increasing BEPI, among SsdA4mix variants with activity above BE3. Data represent mean values of two biological replicates (*n *= 2).

The analysis revealed several high‐performing substitutions: R14: DW / Q17: P / V33: FHWY / K39: AI (Figure 3a). Notably, ring‐structured residues at V33 demonstrated substantial effects on activity, highlighting the importance of this position in active site remodeling (Figure S5). Based on these findings, 90 combinatorial mutants, including WT sequences, were constructed for further optimization (Figure S6). This library, named in SsdA4mix XXXX format (e.g., R14Q17V33K39), forms the basis for subsequent rounds of directed evolution, aiming to identify variants with improved activity and indel formation.

4mix variants demonstrated broader activity across a wider range of sgRNAs compared to individual site‐saturation mutagenesis mutants of the four positions. Using our BEPI evaluation system, we identified 12 superior SsdA4mix variants that outperformed BE3 while remaining below BE4max performance levels (Figure 3b). Among these candidates, we excluded RQFI and WPVA variants due to their elevated indel rates and selected the remaining variants for combination with K31X mutations to identify optimal synergistic combinations.

### 3.7 Combination and Evaluation of SsdA5mix Variants

**FIGURE 4:**
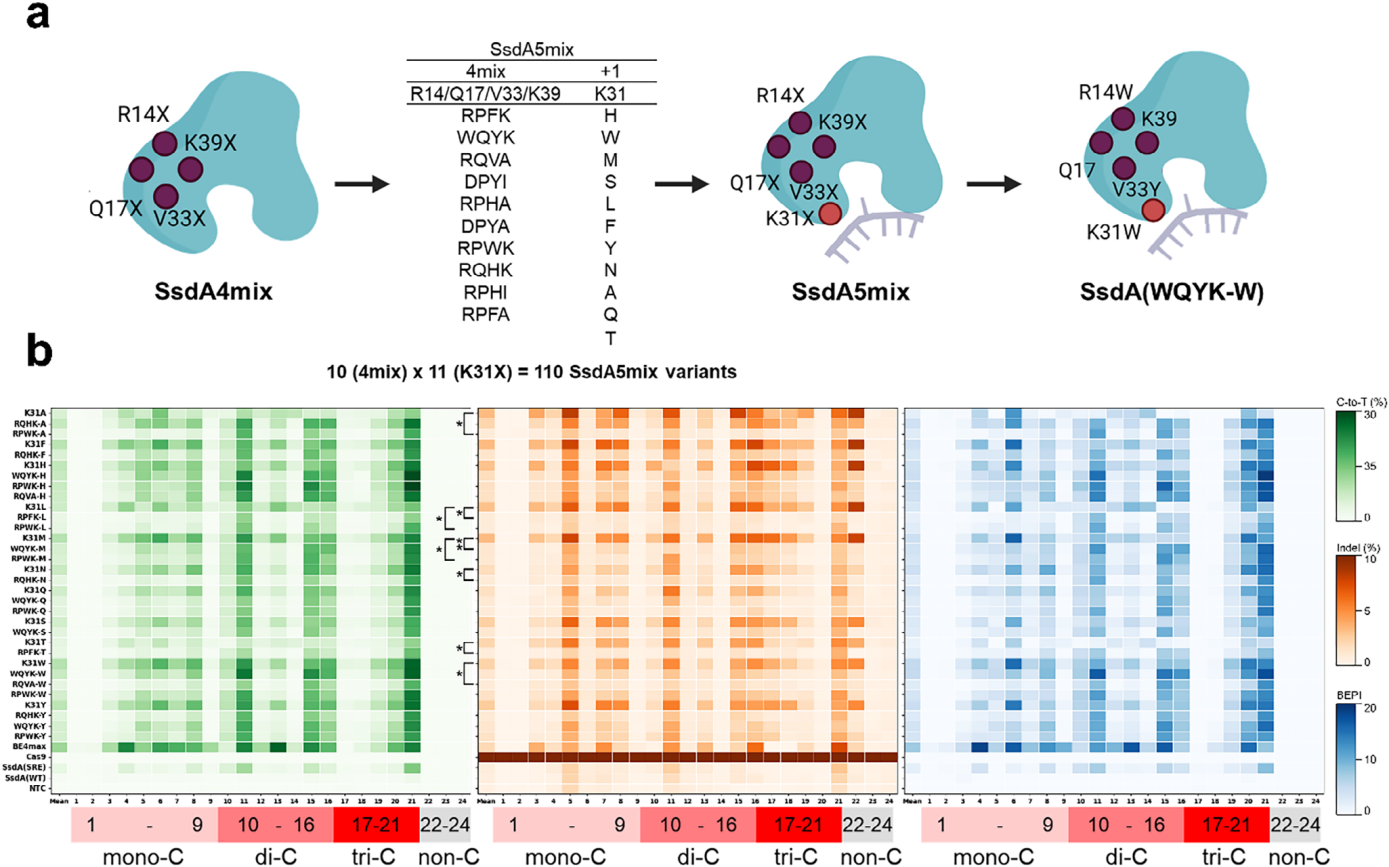
Evaluation and validation of SsdA5mix CBE in exogenous targets. (a) Schematic workflow for SsdA5mix development from SsdA4mix. Top‐performing mutations from SsdA4mix were combined with high‐activity mutations from K31X site saturation mutagenesis. The final selected variants were validated through geometric analysis of their binding pockets. (b) Performance comparison between K31X single variants and top‐performing SsdA5mix variants incorporating each K31X mutation. Performance evaluated using 24 EMsgs with data representing mean values of two biological replicates (*n* = 2). The first column shows mean values across all 24 EMsgs. The *p*‐value for the Indel values obtained from 24 targets with two replicates was calculated using a two‐tailed Welch's *t*‐test (**: *P* ≤ 0.01, *: *P* ≤ 0.05).

To address the trade‐off between activity and indel rates, the 4mix library was integrated with K31X variants, resulting in the 5mix library (XXXX‐K31X) (Figure 4a). This yielded 110 unique mutants, which were systematically evaluated in model HEK293T cells (Figure S7a). Among the 110 mutants, only those that maintained activity while exhibiting reduced indel frequencies were selected for further analysis. To evaluate potential synergistic effects, we compared K31X and SsdA5mix using BEPI heatmaps across the 24 exogenous targets (Figure 4b), and further analyzed C‐to‐T, indel, and BEPI values normalized to BE4max for K31X, SsdA4mix, and SsdA5mix on the same set of 24 targets (Figure S7b). In this representation, variants such as RQHK A, RPWK H, WQYK H, WQYK Y, RPWK Y, RQHK N, and WQYK W show BEPI values that are higher than either the corresponding K31X single mutants or the 4mix backbone alone at multiple sites, indicating that the 4mix mutations can further reduce indel rates or enhance activity on top of the K31X substitutions in a sequence‐dependent manner.

**FIGURE 5:**
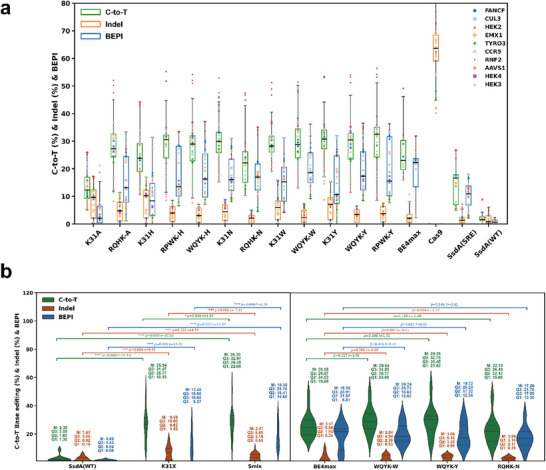
Evaluation and validation of SsdA5mix CBE in endogenous targets. (a) Validation results for selected SsdA5mix variants, their corresponding K31X combinations, and controls (BE4max, Cas9, SsdA(SRE), SsdA(WT)) at 10 endogenous target sites, presented as box plots. The central line within each box denotes the median value, while the whiskers represent data within 1.5 times the interquartile range (IQR). The IQR is defined by Q1 (25th percentile) and Q3 (75th percentile). The lower whisker extends to the greater of the minimum value or Q1 − 1.5×IQR, and the upper whisker extends to the lesser of the maximum value or Q3 + 1.5×IQR. Individual data points are overlaid on each box plot, with different symbols and colors representing different target sites from three independent experiments per target site. (b) Violin plots comparing the performance of SsdA‐based editors across endogenous target sites. Each violin displays the median (Q2), first quartile (Q1), and third quartile (Q3) values. Mean values (M) were additionally calculated to facilitate comparison and plotted on each violin. Statistical significance was determined by *t*‐tests, with *p*‐values and t‐statistics indicated above each comparison. Left: Comparison Among SsdA variants (WT, K31X, and 5mix groups). K31X contains SsdA_tox_ substituted with A, H, N, W, and Y at position 31. The 5mix group contains RQVA‐A, RPWK‐H, RQHK‐H, RQHK‐N, WQYK‐W, WQYK‐Y, and RPWK‐Y variants. Right: Comparison of BE4max with selected SsdA variants (WQYK‐W, WQYK‐Y, and RQHK‐N).

5mix variants were further assessed using HEK293T's endogenous targets (AAVS1, CCR5, CUL3, EMX1, FANCF, HEK2, HEK3, HEK4, RNF2, TYRO3) (Figure 5a). Individual target analysis showed varied performance: compared to K31X, these variants showed similar or improved activity while reducing indels by nearly two‐fold. Compared to SsdA_tox_ (SRE) [34], which exhibited 14.23% C‐to‐T activity, 1.82% indel rate, and 10.34 BEPI, the selected 5mix variants demonstrated substantial improvements. For instance, SsdA(WQYK‐W) achieved 29.64% activity (2.1‐fold increase), 2.94% indel rate (1.6‐fold increase), and 20.24 BEPI (2.0‐fold improvement), indicating that the enhanced activity more than compensated for the modest indel increase. Against BE4max, the performance varied with target sequence: for AAVS1, CUL3, and FANCF, both SsdA5mix and BE4max exhibited low indel rates. However, for CCR5, 5mix variants outperformed BE4max in reducing indels, while EMX1 and HEK3 showed BE4max with significantly higher indel rates (Figure S8). Conversely, BE4max had lower indel rates for HEK2, HEK4, and TYRO3. Notably, RNF2 demonstrated lower indels exclusively with SsdA5mix variants.

These sequence‐dependent activity differences revealed distinct sequence recognition contexts between APOBEC1 and SsdA5mix variants, a pattern consistently observed across both 24 exogenous targets (Figure S9a) and 10 endogenous targets (Figure S9b). While BEPI provides a metric for activity and indel formation, it does not capture other dimensions of base editing specificity. To obtain a more comprehensive view of base editing outcomes, we analyzed all deep sequencing data to quantify three classes of events: (i) total C‐to‐T conversions within the editing window, (ii) C‐to‐T conversions outside this window at the amplicon level, and (iii) all other base substitutions observed within the window (Figure S10). For class (i), BE4max displayed broad activity spanning cytosine positions 6 to 19 relative to the PAM in exogenous targets and positions 10 to 18 in endogenous targets (Figure S10a,b). In contrast, the three optimized SsdA5mix variants (WQYK‐W, WQYK‐Y, RQHK‐N) consistently showed high activity within a narrower window restricted to positions 12 to 17, independent of target class. For class (ii), both BE4max and SsdA_tox_‐based editors generated C‐to‐T conversions outside this defined window, but SsdA_tox_ variants again showed higher specificity, with uniformly lower levels of out‐of‐window C‐to‐T editing.

As an additional analysis, C‐to‐D analysis revealed several informative patterns. In exogenous targets, positions 2, 9, 14, and 17 showed distinct differences between SsdA variants and BE4max despite their overall similarity (Figure S10c). SsdA variants displayed largely consistent profiles, but positions 2 and 9 exhibited notable divergence, both corresponding to a TCT sequence context. This suggests that SsdA variants may benefit from context‐specific engineering to reduce undesired C‐to‐D conversions at TCT motifs. In endogenous targets, BE4max produced the highest C‐to‐D levels at HEK4 and TYRO3 while generating the lowest indel frequencies, whereas the opposite trend was observed at HEK3 (Figures S8 and S10d). These results indicate that C‐to‐D conversion and indel formation are not inherently correlated, highlighting the need to assess these metrics independently when evaluating base editor performance. In addition, differences between exogenous and endogenous target patterns, including shifts in the effective editing windows that are more pronounced for BE4max than for SsdA_tox_, likely reflect locus‐dependent factors. These include the proximity of the U6 promoter and high transcriptional activity in reporter constructs, which may increase single‐stranded DNA exposure, as well as more accessible chromatin at PiggyBac integration sites. Taken together, these observations suggest that BE4max may be more sensitive to changes in the local genomic environment, whereas SsdA_tox_ variants maintain a more stable editing window across contexts, and that both sequence context and chromatin environment modulate specificity at the levels of C to D conversion and indel formation.

Statistical analysis across ten endogenous targets revealed comprehensive performance improvements of SsdA variants (Figure 5b). K31X variants showed substantial activity enhancement from an average of 2.38% to 25.94% (10.8‐fold increase) compared to SsdA_tox_ WT. However, indel rates also increased from 1.61% to 6.58% (four‐fold increase), resulting in approximately 20‐fold improvement in BEPI evaluation. SsdA5mix variants achieved superior performance with average activity of 28.2% (11.8‐fold increase over WT) while reducing indel rates to approximately half those of K31X variants. The final BEPI evaluation showed 28.2‐fold improvement for 5mix variants, demonstrating that the screening‐derived 4mix mutations effectively compensated for the indel limitations of K31X, though additive effects on activity between K31X and 4mix were limited.

Detailed comparison with BE4max revealed distinct performance profiles for the three selected SsdA variants (Figure 5b). Using BEPI as a composite metric, optimized SsdA_tox_ variants showed marked improvements over wild‐type SsdA_tox_ and achieved overall performance comparable to BE4max, with trends toward reduced indel frequencies at many sites, although differences were not statistically significant at all targets. Across the panel of exogenous and endogenous targets tested, SsdA5mix variants and BE4max exhibited similar overall BEPI scores, with SsdA5mix providing favorable trade‐offs between C‐to‐T conversion and indel formation at a subset of sites.

### 3.8 Toxicity Test of SsdACBE Variants Using *E. coli* Transformation System

To evaluate the cytotoxicity of SsdA_tox_ variants, an *E. coli* transformation system was employed. DH5α cells were transformed with pBLC‐SsdACBE variants in the absence of the pRep‐sgRNA to isolate the effects of SsdA_tox_ alone. Without sgRNAs, the transformation reflected the intrinsic toxicity of SsdA_tox_ (Figure S11). The results revealed that wild‐type (WT) SsdA_tox_ exhibited the highest toxicity, forming ten‐fold fewer colonies compared to other mutants. In contrast, variants such as K31N and K31W displayed significantly reduced toxicity, producing many colonies comparable to the control. Similarly, 4mix and 5mix variants also formed numerous colonies, indicating minimal toxicity. The observed reduction in toxicity for K31N and K31W variants can be attributed to their reduced binding affinity for ssDNA, a consequence of the K31 residue's role in facilitating interactions with the ssDNA backbone phosphate. Mutating K31 diminished these interactions, resulting in decreased off‐target effects and toxicity. Interestingly, 4mix variants, which do not directly interact with the ssDNA backbone, also exhibited reduced toxicity. Structural analysis suggests that 4mix mutations enlarge the DNA binding pocket, potentially reducing non‐specific interactions with ssDNA. This reduced non‐specific deamination may explain the decreased cytotoxicity observed in these variants. These findings demonstrate that the high cytotoxicity of WT SsdA_tox_ is mitigated in engineered variants, particularly those incorporating K31 mutations or 4mix/5mix modifications, making them more suitable for base‐editing applications.

### 3.9 Structural Insights Into 5mix Variants

To elucidate the structural basis underlying the performance of engineered variants, comprehensive computational analyses were conducted using AlphaFold3 for structure prediction, followed by inter‐residue distance measurements in MOE. Spatial positioning of key residues was further examined through trilateration analysis (Figure S12).

Structural analysis suggested two distinct optimization patterns in the engineered variants. The critical distances between K31X and the Y76, Y84, and K106 residue triad, which define the pocket entrance, showed minimal changes in K31X single mutants and 4mix variants relative to WT. In contrast, the 5mix variants exhibited markedly increased entrance distances arising from combinatorial substitutions, albeit with distinct specificity patterns. WQYK displayed enhanced distance expansion specifically with W and Y substitutions (WQYK‐W and WQYK‐Y), whereas RQHK showed a similar effect exclusively with the N substitution (RQHK‐N).

While K31W and K31Y single mutants exhibited the greatest distances from the Y76–Y84–K106 triad among the K31X series, the K29 residue in WQYK‐W and WQYK‐Y variants was positioned closest to this same residue set, with substantial distance shifts. This pattern is consistent with a potential compensatory role of K29 relative to K31 in mediating interactions near the phosphate backbone. RQHK‐N, however, displayed a distinct structural arrangement, possibly because its asparagine substitution retains spatial similarity to the WT lysine, suggesting a mechanism different from the K29‐associated compensation observed in WQYK variants (Figure S12b). These structural observations underscore the coordinated architectural rearrangements underlying variant performance and indicate that combinatorial substitutions can modulate the pocket entrance through variant‐specific mechanisms.

## 4 Conclusion

We combined AlphaFold‐guided mutation scan with Trinity‐Screen, a novel *E. coli*‐based directed evolution system, to optimize SsdA_tox_, a compact deaminase from *Pseudomonas syringae*. This integrated approach successfully developed SsdA_tox_ variants that outperform BE4max, demonstrating the value of combining computational and experimental strategies. Although several previous studies have attempted to improve SsdA through rational design approaches, our work represents the first successful development of SsdA variants that outperform BE4max when evaluated as conventional CBE systems using transfection‐based assays [34, 62, 63]. The framework is generalizable to other deaminase enzymes from diverse species, expanding the base editor toolkit.

Notably, none of the mutation sites we identified overlapped with those reported in other recent SsdA engineering studies. The positions enriched by Trinity‐Screen (R14, Q17, V33, K39) together with our rationally nominated K31 (R31/K31X) are distinct from loci highlighted in independent SsdA or DYW‐family efforts [34, 62, 63], underscoring that our selection regime uncovered an orthogonal solution space for improving activity. This mirrors our previous experience with Cas9 specificity engineering, where Sniper‐Cas9 [46] and Sniper2L [13] carried non‐overlapping substitutions relative to other high‐fidelity Cas9s [64, 65, 66, 67].

AlphaFold‐driven alanine scanning identified K31 as the position most likely to affect pocket size and activity. Experimental validation showed that K31X mutations dramatically alter activity, with most amino acid substitutions except K and R showing significant functional changes, as these residues can maintain DNA phosphate interactions. K31X mutations enhanced editing efficiency 10.8‐fold compared to wild‐type. We acknowledge that AlphaFold has general limitations in predicting ΔΔG or functional outcomes of point mutations [38, 39, 40, 41, 42, 43]. Nevertheless, several features likely explain why our AlphaFold‐guided scan was effective here: (i) the effect is local and geometric, removing the positively charged, bulky K31 side chain widens the ssDNA entrance, a first‐order steric/electrostatic change that AlphaFold can represent; (ii) we quantified simple geometric readouts on DNA‐bound models (entrance distance/accessible area) rather than ΔΔG or activity predictions, aligning with AlphaFold's strength in providing plausible single‐state coordinates); (iii) SsdA_tox_ is compact and appears less conformationally plastic, reducing the influence of unmodeled alternative states; and (iv) the in‐silico scan served only as a prioritization filter, with empirical selection validating true positives. Together, these considerations rationalize why AlphaFold‐guided alanine scanning was instrumental here, while we remain cautious about generalizing mutation‐effect predictions.

To our knowledge, this is the first validated instance in a genome‐editing enzyme where AlphaFold3‐guided alanine scanning directly led to a gain‐of‐function single‐site design. We speculate that AlphaFold3's improved handling of DNA–protein complexes enabled meaningful assessment of pocket‐entrance geometry in a bound state. Even so, experimental verification remains essential, as static structure predictions cannot anticipate complex functional side effects intrinsic to deaminases.

Supporting our experimental findings, a recently published crystal structure independently confirmed K31's role in phosphate backbone interactions, with the authors identifying the K31 position (K289 in their numbering) as making critical interactions with ssDNA [62]. However, without crystal structures for K31 mutations available, they simply argued that site‐directed mutation to alanine would disrupt backbone interactions and reduce activity. This explanation does not account for the larger cavity created by displacement of the side chain, a phenomenon that can only be accurately predicted when mutant structures are available.

Despite improved activity, K31X variants presented a significant challenge with elevated indel rates (four‐fold increase), necessitating further optimization. For example, among K31X variants, K31I and K31V displayed such pronounced toxicity in Escherichia coli that we were unable to recover plasmids bearing these substitutions after transformation. During multiple independent cloning attempts, colonies that grew consistently harbored frameshift mutations at codon 31 rather than the intended missense substitutions, indicating strong selection for loss of SsdA function. Similar recurrent frameshift events have been described as a bacterial escape route when plasmids encode toxic gene products and are widely used as a proxy for gene toxicity in *E. coli* [68, 69]. Isoleucine and valine are non charged residues similar to other high activity substitutions at this position such as W, N, and M, in contrast to low activity charged residues such as D, K, and R. Together with the observation that K31I and K31V were toxic even in the absence of a guide RNA, these findings suggest that their effects are not restricted to on target deamination but instead reflect hyperactive deaminase behavior with extensive non specific deamination of DNA and possibly RNA. At present, we cannot resolve the relative contributions of DNA versus RNA off‐target editing to this toxicity, and future genome‐wide and transcriptome‐wide profiling will be required to determine whether K31I and K31V primarily enhance DNA activity, RNA editing, or both. More broadly, these data support a model in which the cytotoxicity of SsdA variants in *E. coli* is linked to the overall burden of global deamination rather than to on‐target activity alone.

In addition, Trinity‐Screen effectively eliminated high‐DSB variants during evolution, demonstrating the power of our triple‐layered screening approach. The resulting 5mix variants achieved 11.8‐fold activity increase with indel rates reduced to half those of K31X variants. AlphaFold‐based structural analysis revealed how 4mix and 5mix mutations address the K31X indel problem through cooperative optimization. These mutations work synergistically to maximize both tunnel mouth accessibility and interior contraction. Specifically, in the 5mix variants, particularly WQYK‐W and WQYK‐Y combinations, K31X mutations increase the distance from both Y76 and K106, widening the tunnel mouth, while K29 undergoes contraction toward both Y76 and K106 to compensate for the original K31 function. This pronounced architectural reorganization in 5mix variants explains their superior performance, where the contracted pocket volume contributes to reduced indel formation through tighter substrate control, while the widened tunnel mouth ensures efficient cytosine access.

Using our BEPI evaluation system, we systematically ranked all SsdA variants against BE4max, enabling comprehensive performance comparison across multiple parameters. We identified variants with distinct performance profiles. SsdA(WQYK‐W) showed 9% better overall performance than BE4max, SsdA(WQYK‐Y) demonstrated 6% improvement with the highest activity, and SsdA(RQHK‐N) exhibited 37% lower indel rates despite slightly reduced activity. These variants provide tailored options for different applications SsdA(WQYK‐W) and SsdA(WQYK‐Y) for scenarios requiring high editing efficiency, and SsdA(RQHK‐N) for applications where minimal indel formation is critical. While activity synergy between K31X and 4mix was limited, 4mix mutations effectively compensated for K31X indel limitations through the sophisticated pocket remodeling mechanism. The BEPI framework extends beyond editing‐indel relationships and can be applied to any scenario where effects and side effects coexist, such as on‐target versus off‐target activity comparisons. The adjustable weighting system for side effect parameters makes BEPI a versatile tool for comprehensive performance evaluation across diverse biotechnological applications.

Because the ultimate goal of base editors is the precise correction of pathogenic alleles in patients, we interpret BEPI in the broader context of prior efforts to jointly assess editing efficiency and mutational safety profiles, encompassing genome‐wide off‐target events, bystander editing, out‐of‐window edits, and noncanonical substitutions beyond C‐to‐T in mammalian systems [10, 11, 53, 70, 71, 72]. These and related studies consistently show that genome‐wide off‐target activity primarily arises from Cas9 nickase or dCas9 binding to mismatched genomic sites rather than from the deaminase module itself [11, 73, 74]. We and others have developed Cas9 variants with improved targeting specificity that, when incorporated into base editors, markedly enhance their editing precision by reducing off‐target activity at mismatched genomic sites [24, 46, 75]. In our prior work, we employed the term ‘specificity’ or ‘specificity ratio’‐ defined as the ratio of on‐target to off‐target editing efficiency‐ as a guiding metric to engineer and identify variants that simultaneously maintain high activity and improved specificity [13, 46]. This quantitative approach enabled the selection of Cas9 backbones that not only preserve robust base editing at intended loci but also minimize unwanted edits at off‐target sites, thereby substantially reducing the genome‐wide mutational burden when these variants are used in cytidine or adenine base editors. Additionally, recent work has shown that rationally designed deaminases can minimize bystander editing, further improving editing precision [76]. While these advances address different aspects of base editing fidelity, our study focused specifically on improving on‐target C‐to‐T conversion efficiency while simultaneously reducing DSB‐linked indel formation. BEPI was developed to describe this relationship: it provides a concise, quantitative measure that integrates on‐target activity with indel burden at a given site, serving as a practical index for comparing variants within the context of our optimization framework. Although BEPI does not explicitly account for bystander activity or genome‐wide off‐target edits, which remain essential considerations for therapeutic development, it is an informative screening tool when complemented by orthogonal assays that assess Cas9‐driven off‐target activity and the full spectrum of editing outcomes.

Beyond catalytic performance, we examined editing specificity across different genomic contexts. The distinct editing windows between APOBEC1 and SsdA variants revealed interesting differences in target specificity. BE4max showed broader windows in exogenous (positions 6–19) versus endogenous targets (positions 10–18), while SsdA(WQYK‐W) maintained consistent windows (positions 12–17) regardless of target type. These differences may reflect differential chromatin sensitivity, as exogenous targets likely have weaker chromatin formation, though this hypothesis requires further investigation. The consistent editing window of SsdA variants suggests potentially more predictable performance across diverse genomic contexts.

This work demonstrates that a systematic AlphaFold‐guided mutation scan combined with high‐throughput directed evolution can effectively overcome the limitations of compact alternative deaminases for base editing. The integration of computational prediction with experimental validation through Trinity‐Screen's triple‐layered selection provides a scalable and mechanistically informed framework for base editor optimization. Beyond SsdA_tox_, this approach is generalizable to other deaminase enzymes from diverse species, establishing a blueprint for developing improved genome editing tools with enhanced precision and reduced side effects.

## Author Contributions

Conceptualization, J.K.L., and R.G.S.; methodology, R.G.S.; investigation, R.G.S., and G.K.; writing – original draft, J.K.L., and R.G.S.; writing – review & editing, J.K.L., and R.G.S.; supervision, J.K.L.

## Conflicts of Interest

A patent application has been filed based on this work: Toolgen filed, Inventor: R.G.S. and J.K.L. covering Trinity‐Screen and SsdA_tox_ variants in this paper.

## Supporting information




**Supporting File**: advs74127‐sup‐0001‐SuppMat.docx.

## Data Availability

We have submitted the deep sequencing data from this study to the NCBI Sequence Read Archive under accession number PRJNA1203172. We have provided the datasets used in this study as Supplemental Dataset 1. Materials described in this study are covered by an issued patent and are available upon reasonable request under a material transfer agreement. The p‐values were calculated using Welch's t‐test and ANOVA t‐test in Microsoft Excel and BioRender. Graphs were generated using BioRender, Microsoft Excel, and Python modules, including pandas, matplotlib, seaborn, and openpyxl.
