# Supplementary material for: Engineering Compact Base Editors by AlphaFold‐Guided Mutation Scan and Escherichia coli‐Based Tri‐Selection
Source: Adv Sci (Weinh). 2026 Feb 3;13(20):e16213. doi: 10.1002/advs.202516213 (PMC13067763; doi:10.1002/advs.202516213)

**
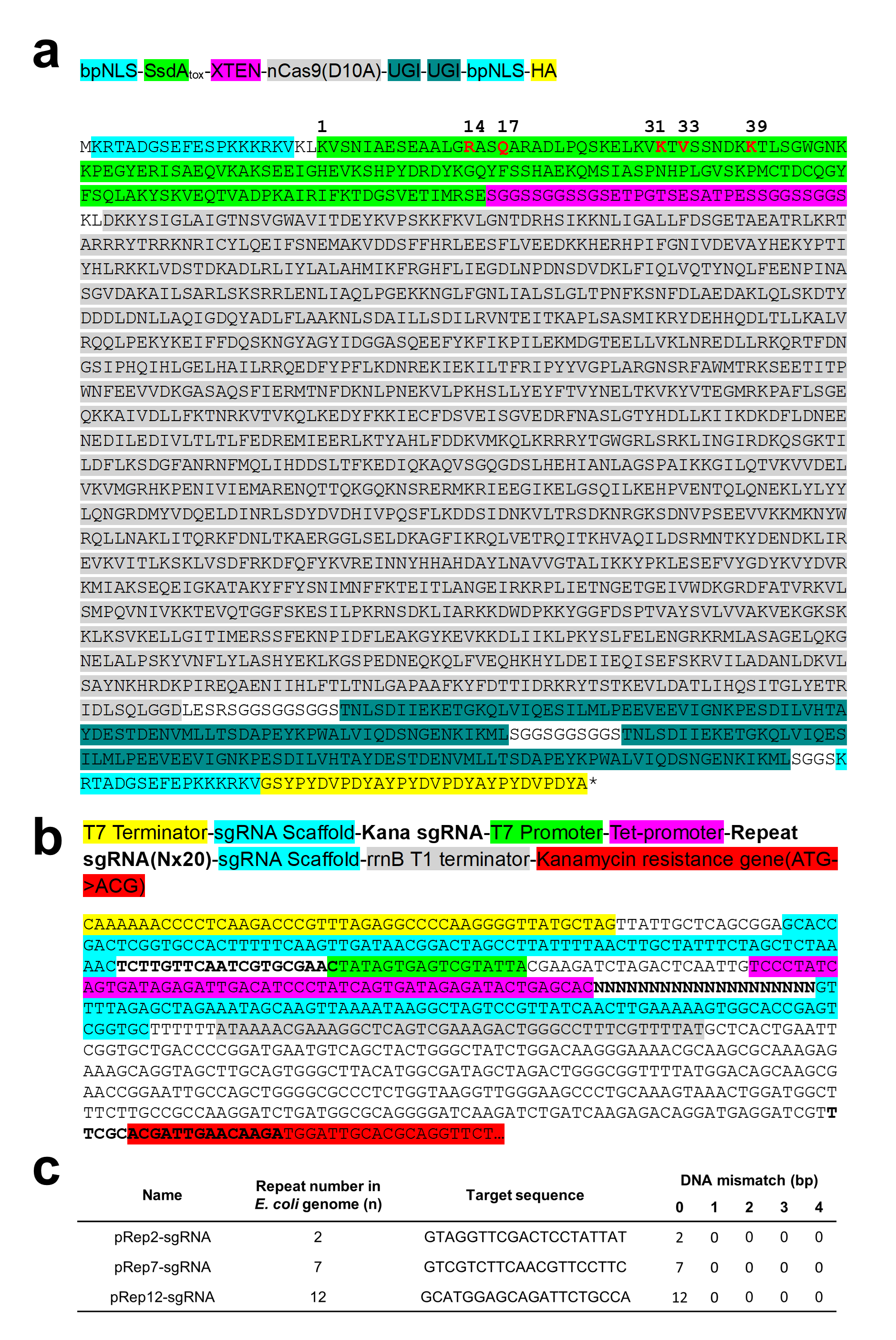
**

**Fig.S1.** **Constructed pBLC-SsdACBE and sgRNA cassettes in this study**. **a,** Sequence information of the SsdA_tox_-based base editor designed in the pBLC plasmid for precise C-to-T base editing, with its modular structure annotated by colors. **b,** Sequence information of two single guide RNAs in the pRep-sgRNA, with modular structures annotated by colors. The sgRNA targeting the mutated start codon of the kanamycin resistance gene is indicated in bold. **c,** Sequence list for targeting the repeat sequence within the *E. coli* genome by repeat sgRNAs, as shown in panel **b**.

**
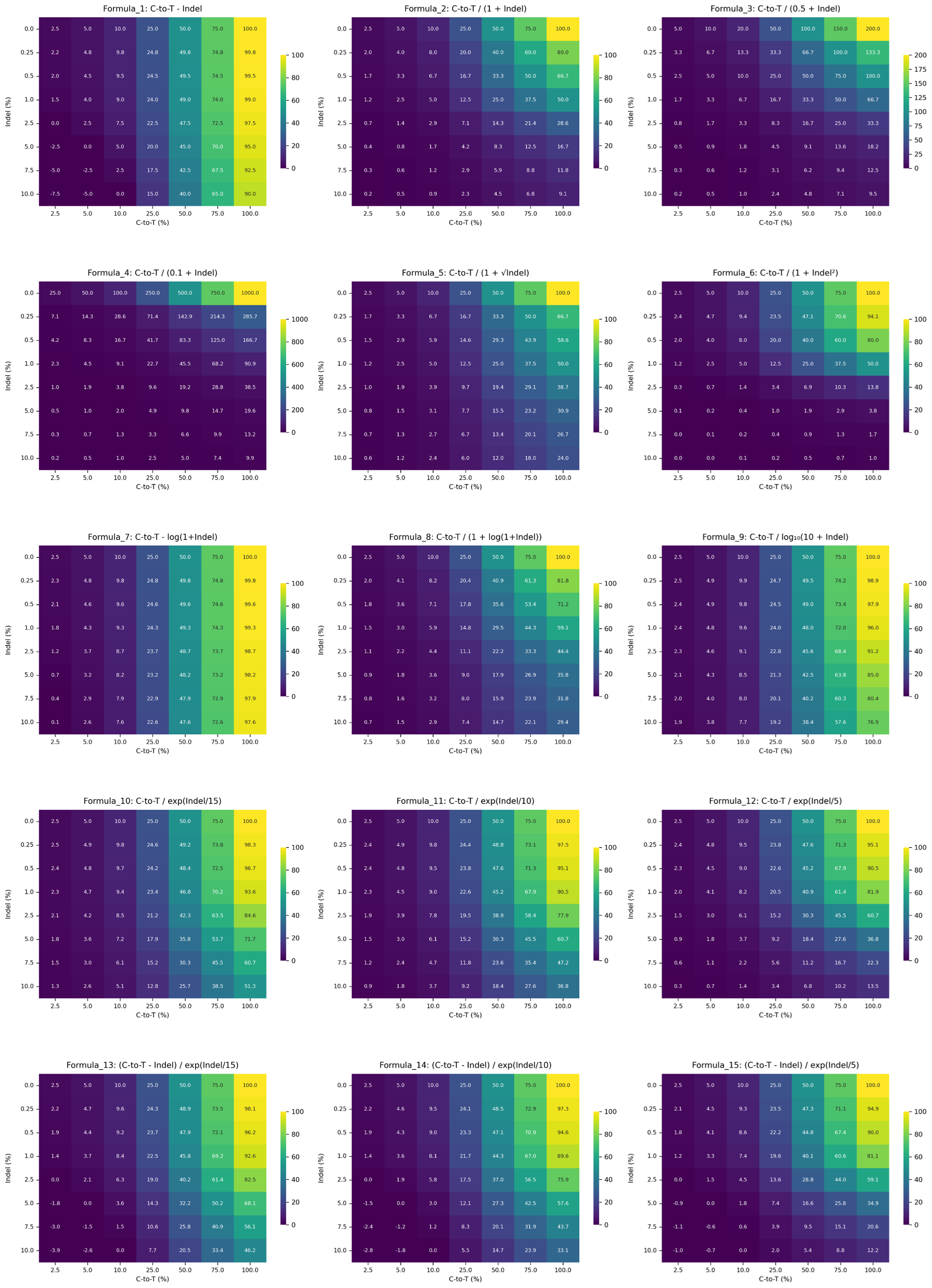
**

**Fig.S2. Development of BEPI models for base editor performance assessment.** Heatmaps showing BEPI values across different C-to-T editing rates (2.5-100%) and indel frequencies (0-10%). Each model represents a different mathematical approach to balance editing efficiency against unwanted indel formation. Yellow indicates higher BEPI values (better performance), while purple/blue indicates lower values.

**
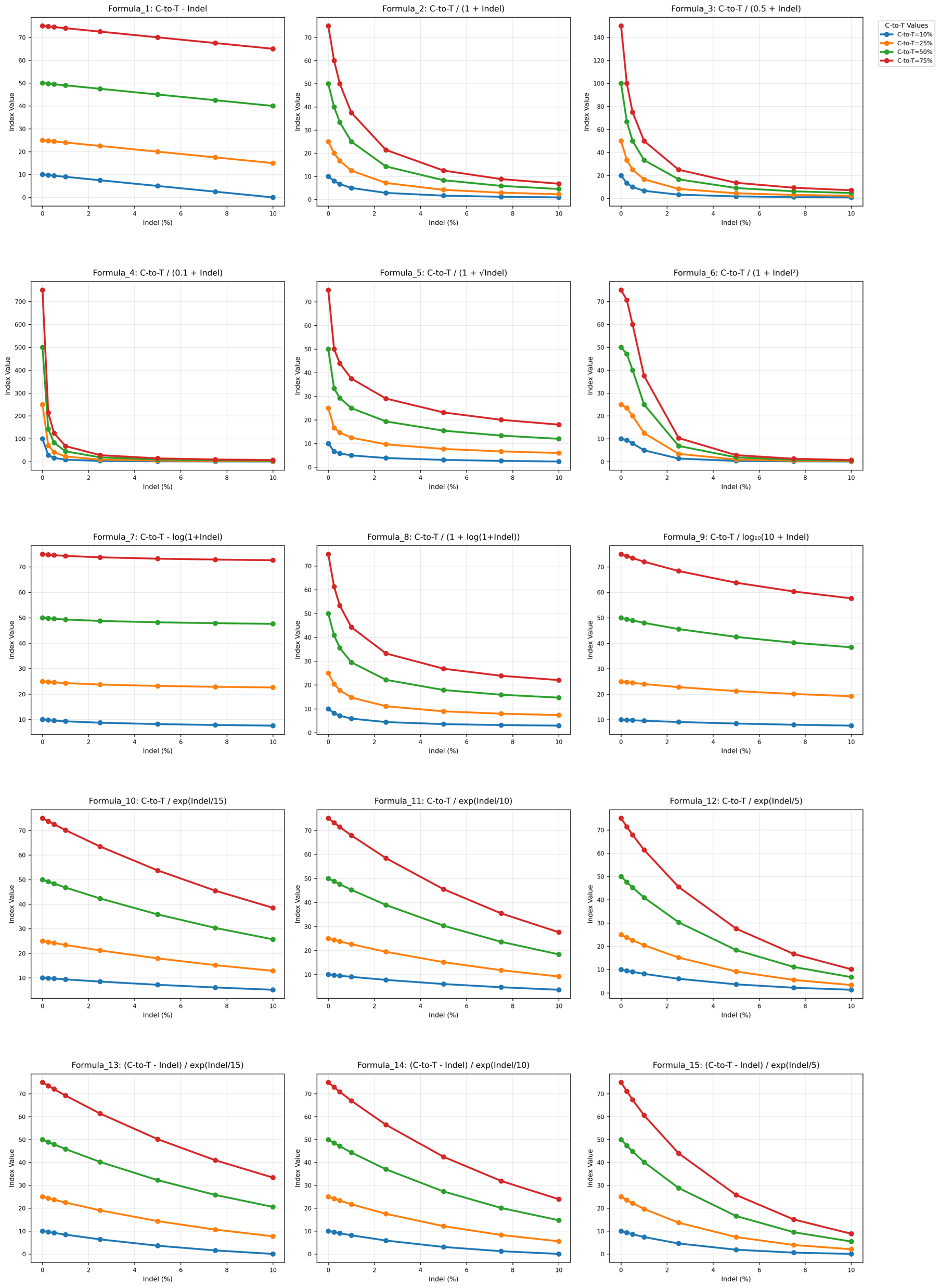
**

**Fig.S3. Sensitivity analysis of 15 BEPI models to indel frequency changes.** Individual plots show how different formulations respond to rising indel rates (0-10%) at four C-to-T conversion levels (10%, 25%, 50%, 75%).


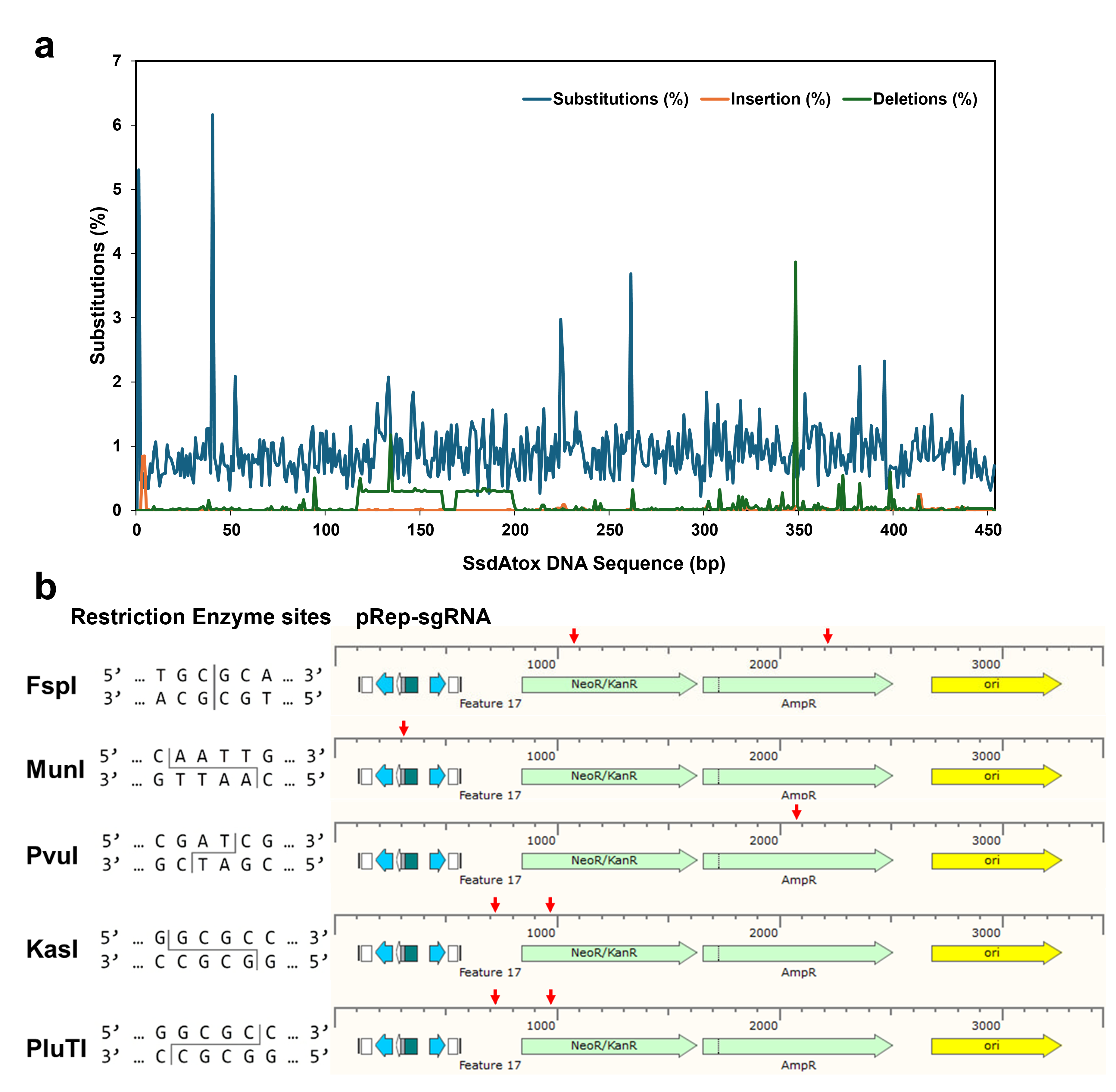


**Fig.S4. Overview of Library Quality and Trinity-Screen Plasmid Design. a,** Substitution and indel frequency across the SsdA_tox_ coding region from the SsdA_tox_ library. **b,** Restriction enzyme digestion map of the Trinity screening pRep-sgRNA. Red arrows indicate restriction enzyme cleavage sites. This pRep-sgRNA design allows recovery of the pBLC plasmid without gel purification in the Trinity screening system. The map was generated using SnapGene.


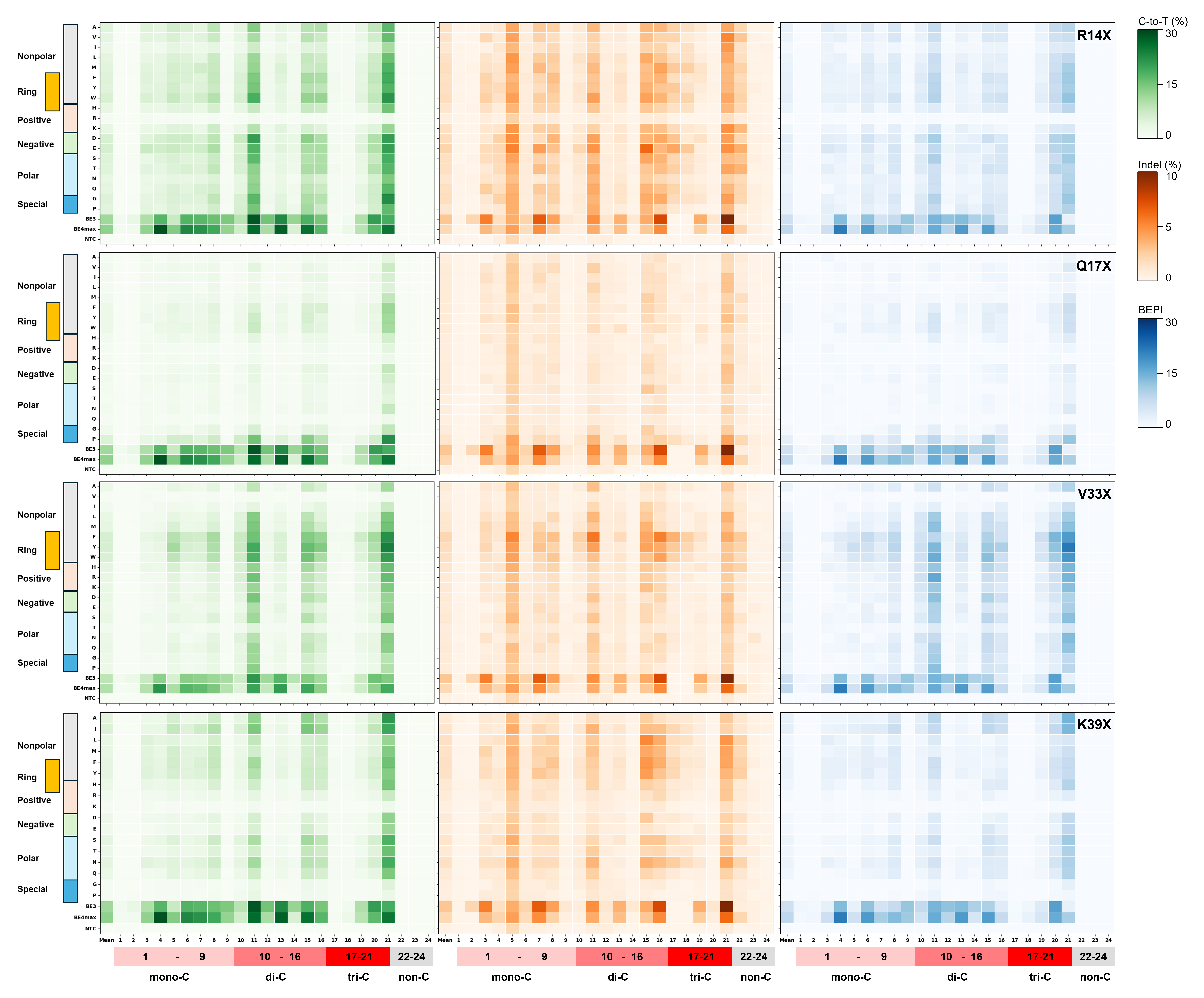


**Fig.S5. Base editing efficiency evaluation of site-saturated residues identified through Trinity screening.** Residues identified through the Trinity screening system were subjected to site-saturation mutagenesis. Base editing efficiency of the resulting variants was evaluated using pooled HEK293T cell library with 24 exogenous sgRNA and target pair. Variants R14X, Q17X, V33X, and K39X were constructed, except for K39V and K39W, which were excluded due to sequence toxicity. Data represent mean value of two biological replicates (*n*=2). The first column in the heatmap represents the mean values across the 24 EMsgs.


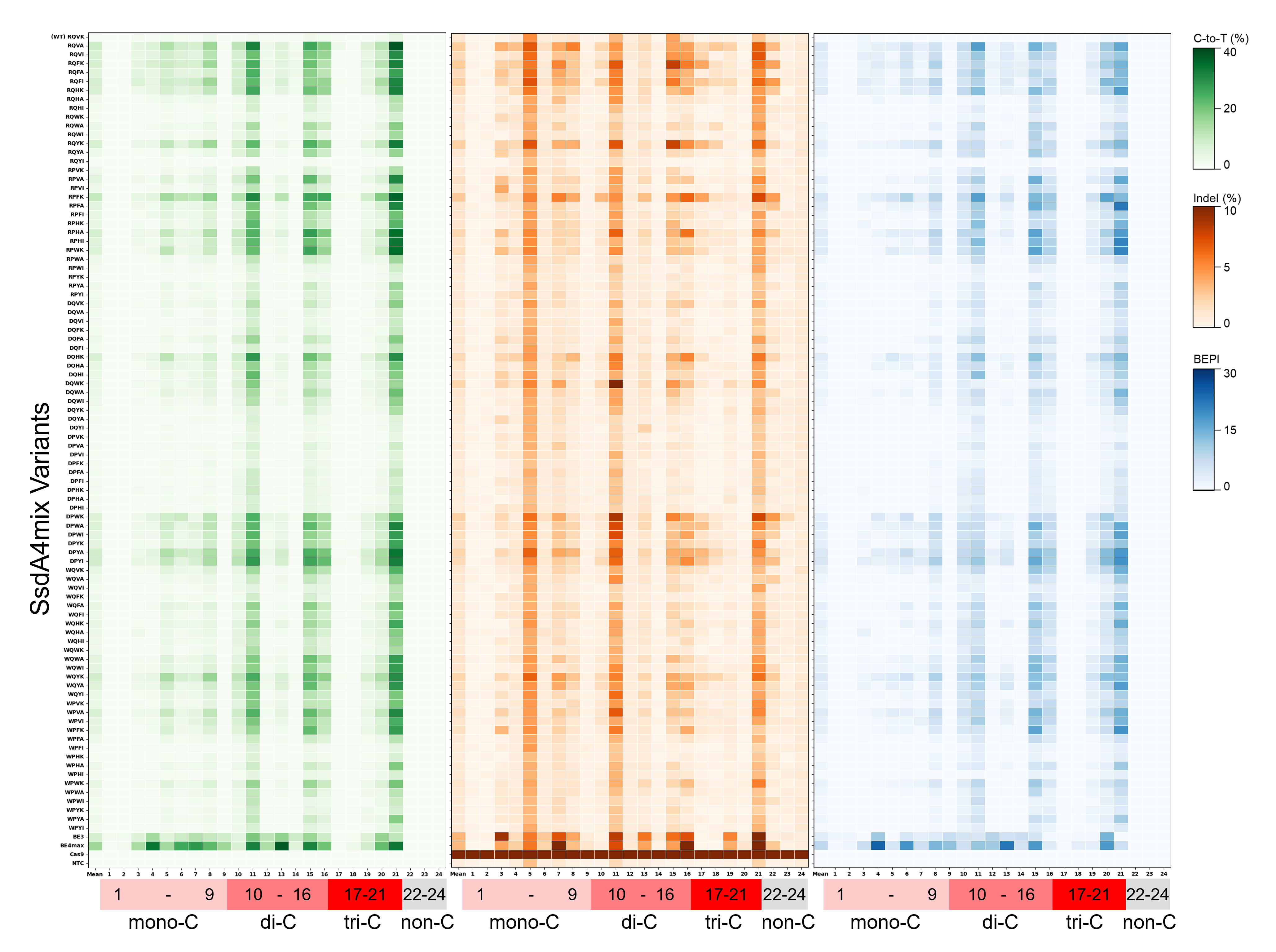


**Fig.S6. Assessment of SsdA4mix variants using 24 EMsgs in HEK293T cells.** Data represent mean value of two biological replicates (*n*=2). The first column in the heatmap represents the mean values across the 24 EMsgs.


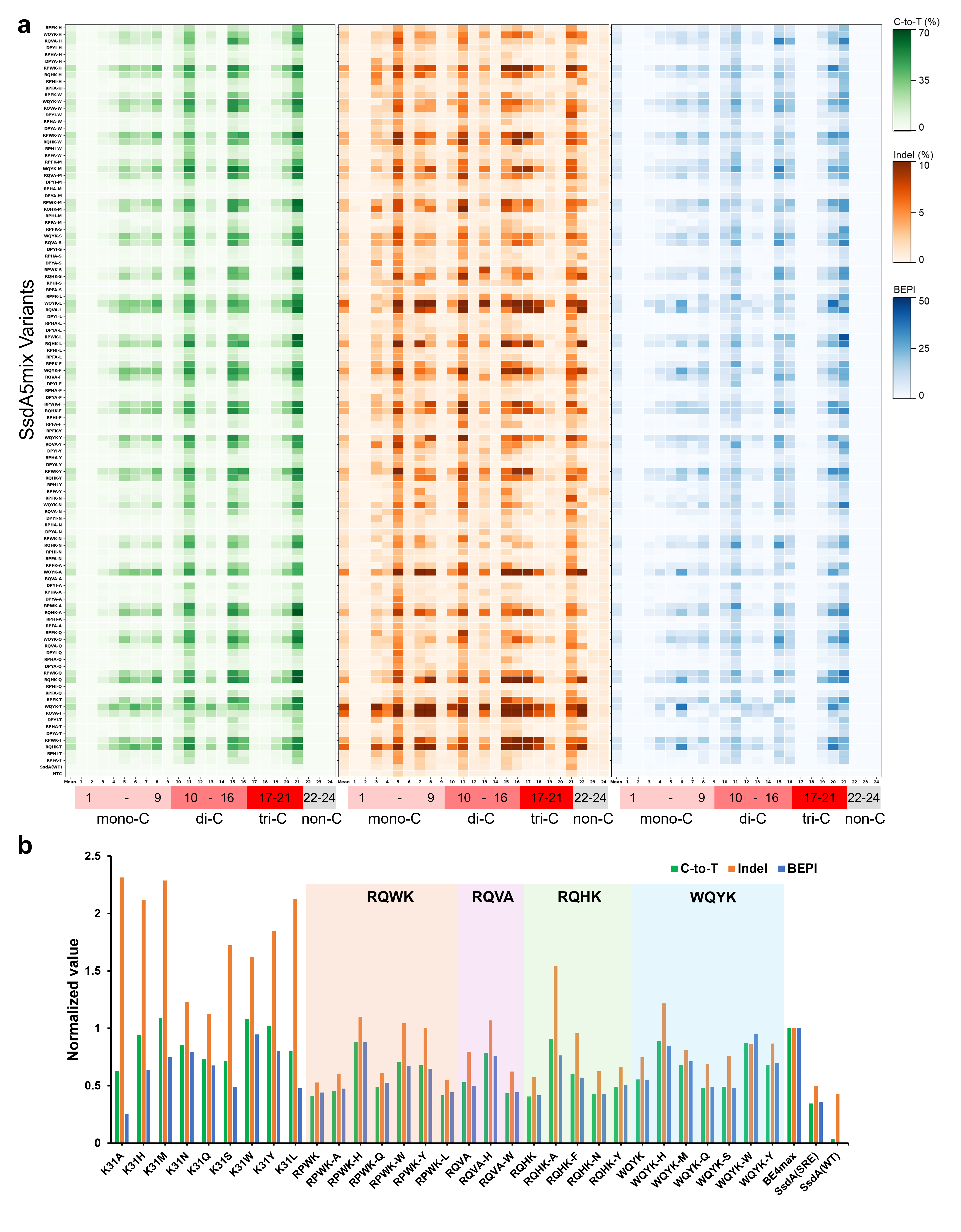


**Fig.S7. Screening of high performance SsdA5mix variants using 24 EMsgs in HEK293T cells.** **a,** Data represent mean value of single biological replicates (*n*=1). The first column in the heatmap represents the mean values across the 24 EMsgs. **b,** C-to-T, indel, and BEPI values for K31X, 4-mix, and 5-mix variants were each normalized to BE4max and plotted using mean values from the 24 EMsgs dataset.

**

**

**Fig.S8**. **Comparative base editing performance at different endogenous target sites.** Data are presented as mean values with error bars representing standard deviation (*n*=3). The left Y-axis represents the C-to-T substitution rate and BEPI values, showing the efficiency of cytosine deamination and overall editing performance at target sites. The right Y-axis depicts Indel rates to evaluate unintended editing outcomes. For Cas9 variants with exceptionally high Indel rates, the graph is partially truncated to enhance visualization clarity and focus on relevant data ranges.

**
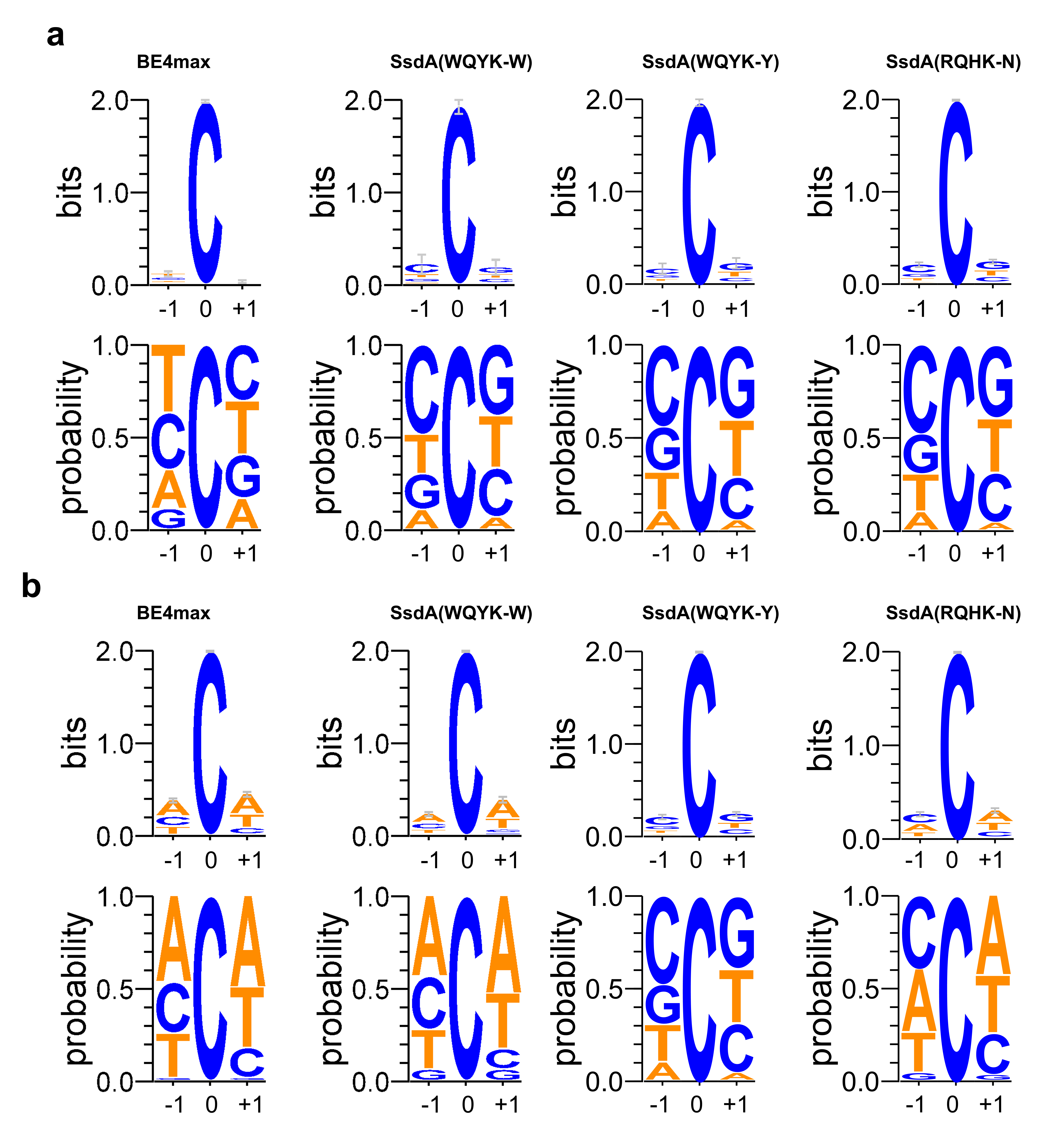
**

**Fig.S9. Sequence context analysis of BE4max and SsdA 5mix variants.** Sequence context analysis was performed by generating combinatorial sequences of nucleotides flanking the target C, creating a FASTA file where each sequence was repeated a number of times proportional to 10× its editing rate, and analyzing the results using WebLogo v3.7.12. Base editing data were collected from **a,** 24 EMsgs and **b,** 10 endogenous targets.

**
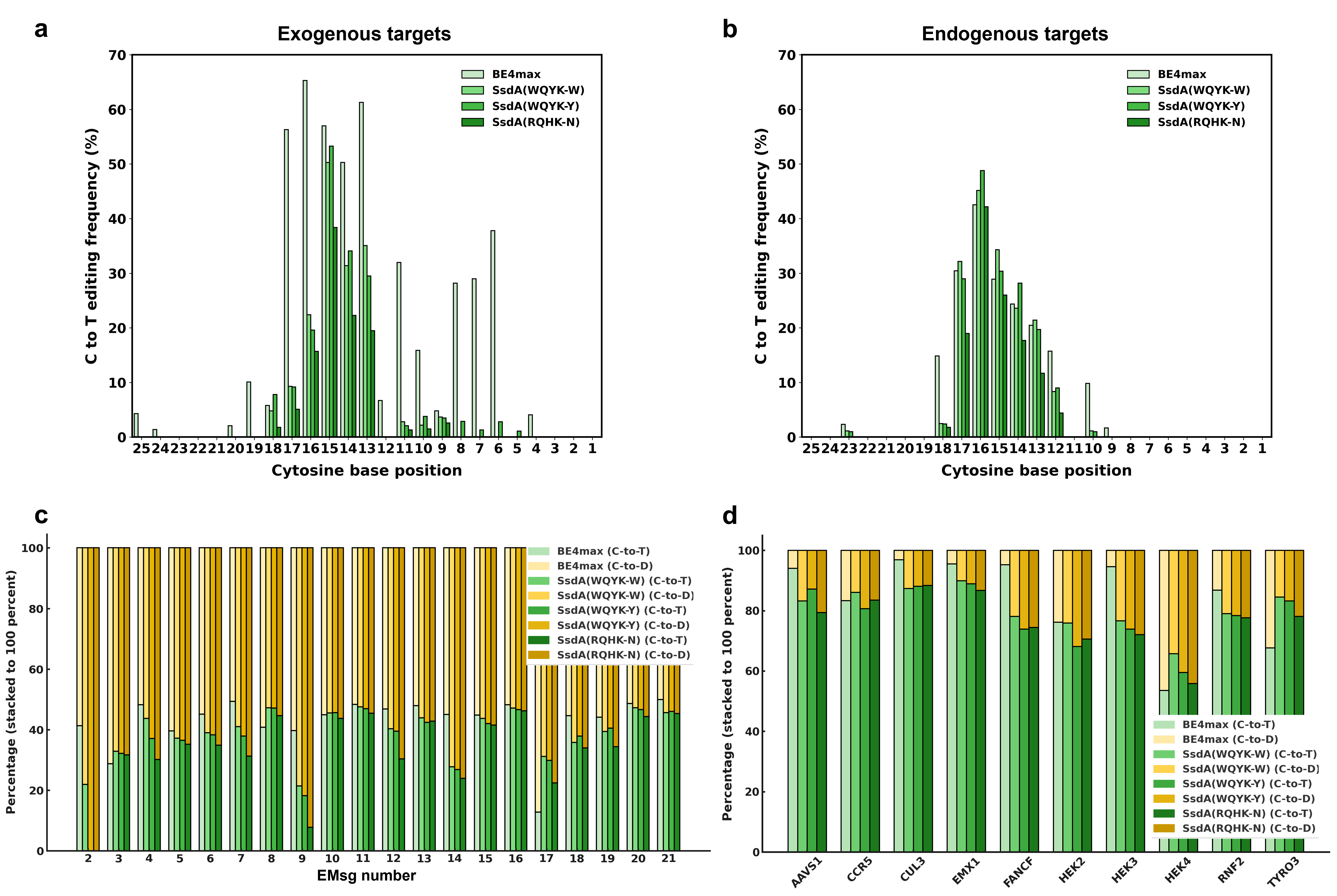
**

**Fig.S10. Comprehensive Profiling of Base Editor Editing Windows and C-to-D Conversion Outcomes. a, b,** Comparison of base editing windows between BE4max and SsdA 5mix variants. Maximum C-to-T editing efficiency at each cytosine position. For each position, the highest average value was selected from multiple sequences containing cytosine at that position. Cytosine positions and editing rates were collected from **a,** 24 EMsgs analyzed in duplicate and **b,** 10 endogenous targets analyzed in triplicate. **c, d,** Quantification of undesired C-to-D conversions across four SsdA variants and BE4max. For each editor–target pair, three independent replicates were performed, and mean values are shown. Bars represent the percentage contribution of C-to-D events relative to the total editing output (C-to-T + C-to-D), displayed as 100 percent stacked columns. C-to-T and C-to-D rates were collected from **c,** 24 EMsgs analyzed in duplicate and **d,** 10 endogenous targets analyzed in triplicate.


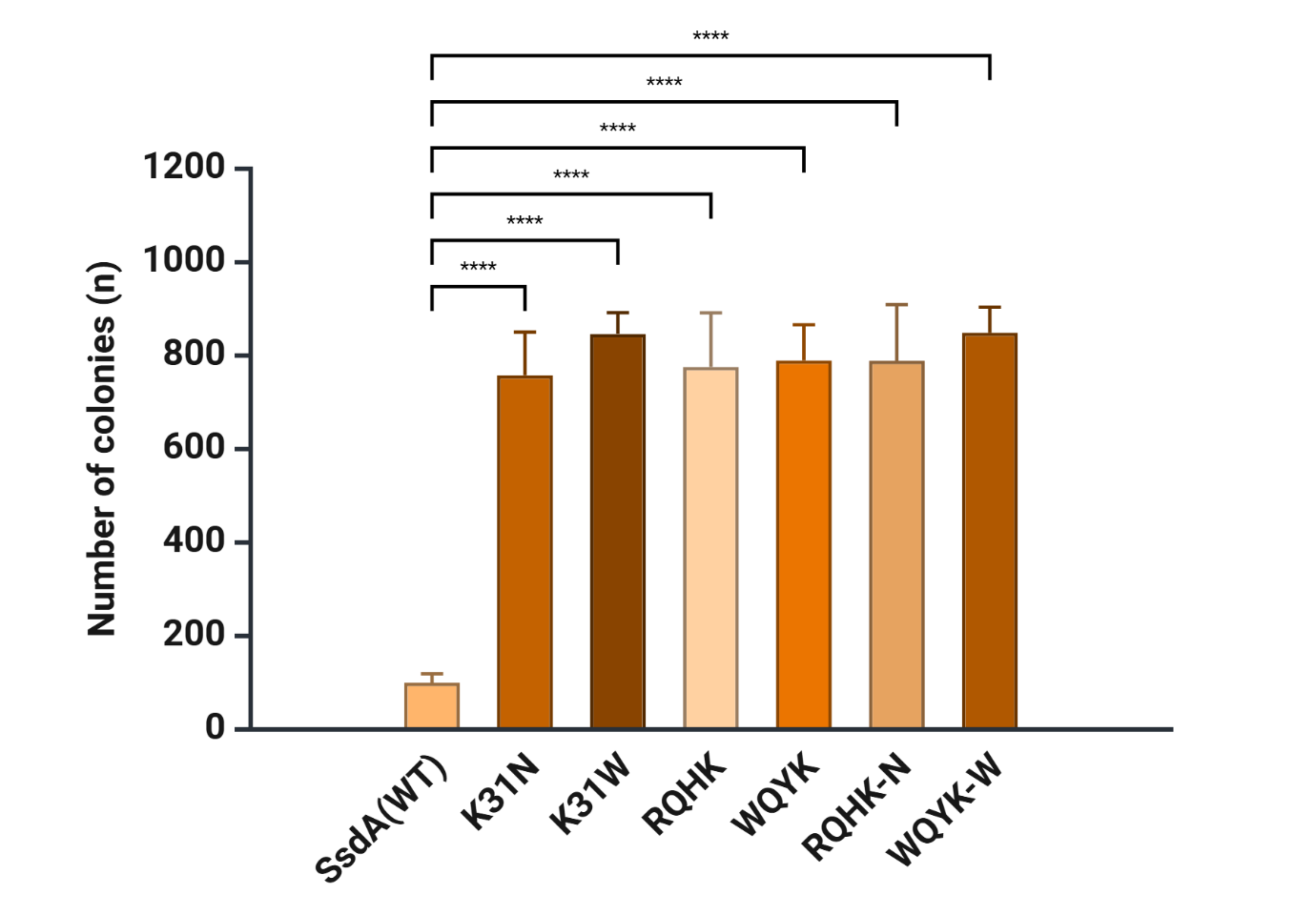


**Fig.S11.** **Bacterial toxicity assay of SsdACBE variants.** DH5α *E. coli* cells were transformed with each SsdACBE variant and plated on chloramphenicol-containing LB medium for colony counting. Bars and error bars represent mean ± standard deviation of biological replicates. Statistical significance was determined by two-tailed Welch's t-test (*n*=3) (****: *p* ≤ 0.0001).

**
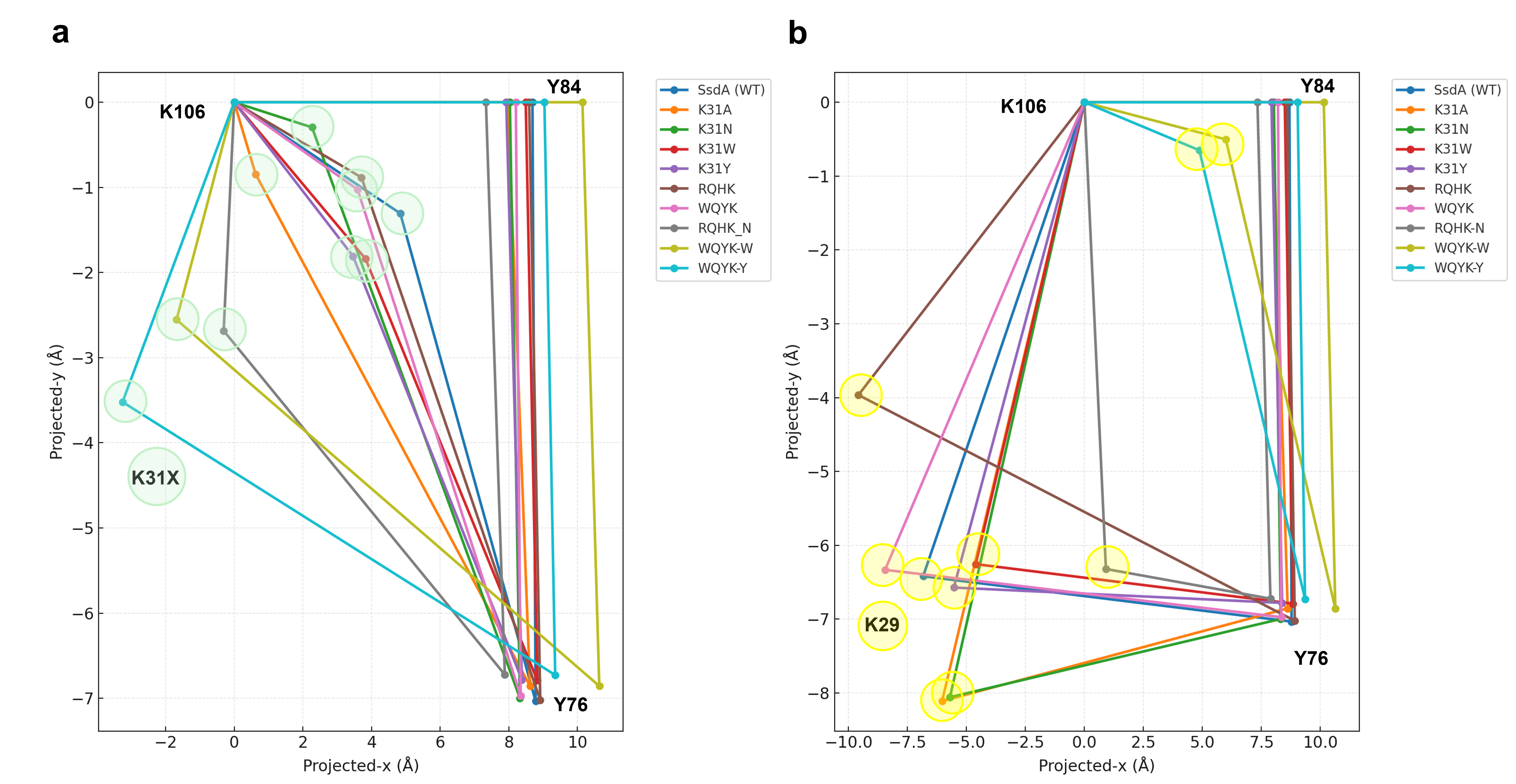
**

**Fig.S12. Geometric analysis of SsdA_tox_ variant structures generated by AlphaFold3. a,** Two-dimensional projected coordinate representation of key residues derived from trilateration analysis. Inter-residue distances were measured between the terminal atoms of amino acid side chains, and all structural analyses were based on averaged values from five AlphaFold3-predicted models. Pairwise distances among the four residues (K31X, Y76, Y84, and K106) were first used to generate three-dimensional coordinates via trilateration. To resolve mirror-image ambiguity and consistently highlight positional differences, the coordinate system was anchored by placing K106 at the origin and aligning the K106–Y84 vector along a fixed axis. These 3D coordinates were then orthogonally projected onto the plane defined by the residue constellation to facilitate clearer visualization of positional variation across variants. K31X positions are marked with green circles in the 2D representation. **b,** Two-dimensional projected coordinate representation of the four residues (K29, Y76, Y84, and K106) from SsdA variants. The same trilateration procedure and projection method were applied to generate the 2D coordinates. K29 positions are marked with yellow circles.

**Table S1**. **Overview of exogenous target sgRNAs and target sequences (EMsgs) integrated into HEK293T cells for high throughput base editing activity assessment.** The red-shaded area represents the 11-19 bp window analyzed by BE-analyzer, highlighting the primary editing window. Cytosines **c,** outside the analysis window are marked yellow, and in cases where no cytosines are present within the 11-19 bp window, the nearest cytosine outside the window is highlighted in blue for reference. This color-coded annotation aids in visualizing cytosine distribution and prioritizing potential base editing targets. **
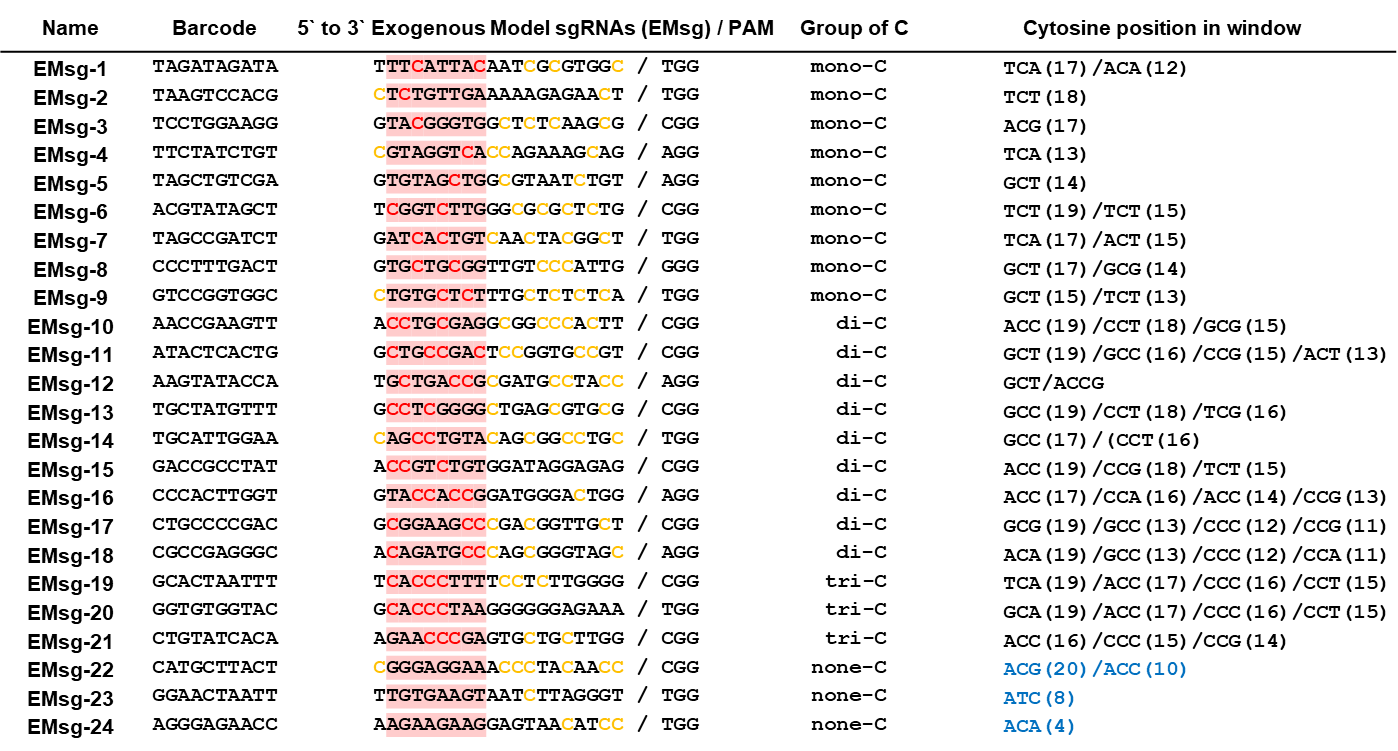
**

**Table S2.** **Mathematical formulas for BEPI.** Fifteen mathematical formulas using C-to-T editing efficiency and indel formation rate to calculate BEPI for comprehensive assessment of activity vs side effect. Formulas are categorized into four mathematical frameworks: arithmetic operations (Formula 1), rational functions (Formulas 2-6), logarithmic scaling (Formulas 7-9), exponential decay models (Formulas 10-12), and enhanced exponential models with direct indel subtraction (Formulas 13-15).


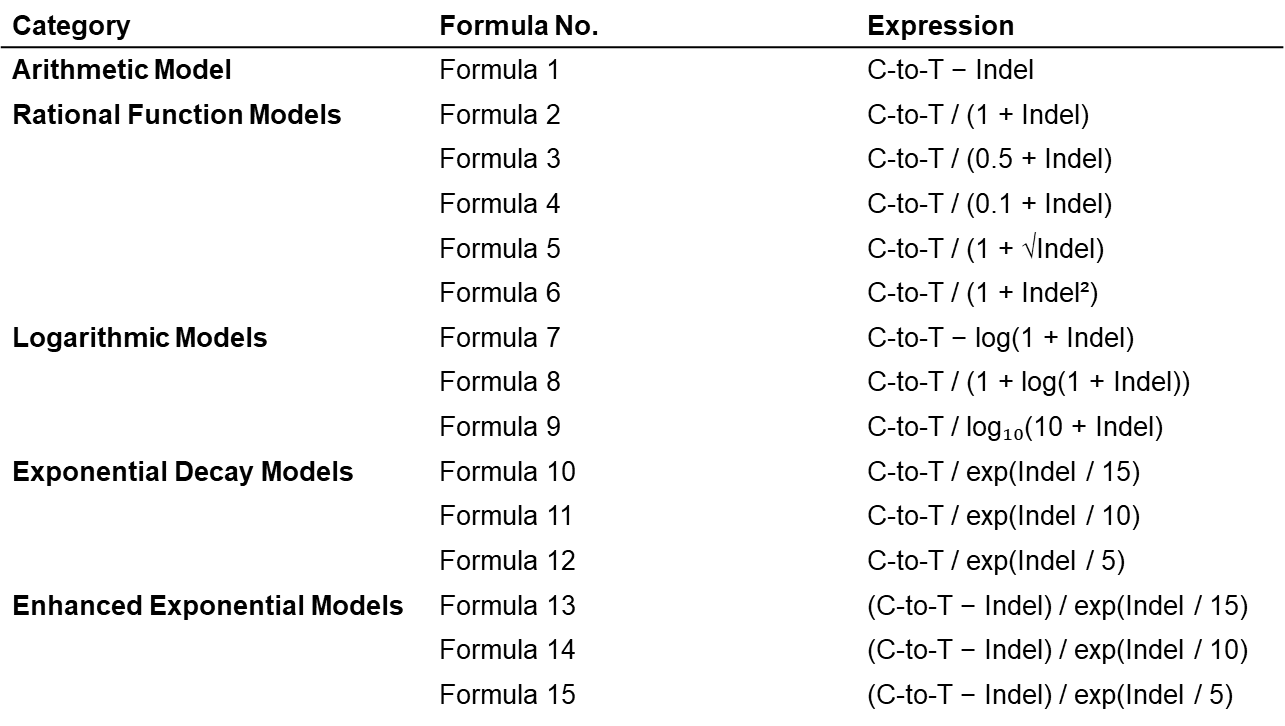

Supplement: Supplementary file 1 — Supporting File: advs74127‐sup‐0001‐SuppMat.docx. [file ADVS-13-e16213-s001.docx]
